# Analysis of SNPs and Haplotypes in Vitamin D Pathway Genes and Renal Cancer Risk

**DOI:** 10.1371/journal.pone.0007013

**Published:** 2009-09-15

**Authors:** Sara Karami, Paul Brennan, Philip S. Rosenberg, Marie Navratilova, Dana Mates, David Zaridze, Vladimir Janout, Helena Kollarova, Vladimir Bencko, Vsevolod Matveev, Neonila Szeszenia-Dabrowska, Ivana Holcatova, Meredith Yeager, Stephen Chanock, Idan Menashe, Nathaniel Rothman, Wong-Ho Chow, Paolo Boffetta, Lee E. Moore

**Affiliations:** 1 Division of Cancer Epidemiology and Genetics, National Cancer Institute, National Institutes of Health (NIH), Department of Health and Human Services (DHHS), Bethesda, Maryland, United States of America; 2 International Agency for Research on Cancer, Lyon, France; 3 Department of Cancer Epidemiology and Genetics, Masaryk Memorial Cancer Institute, Brno, Czech Republic; 4 Institute of Public Health, Bucharest, Romania; 5 Institute of Carcinogenesis, Cancer Research Centre, Moscow, Russia; 6 Department of Preventive Medicine, Faculty of Medicine, Palacky University, Olomouc, Czech Republic; 7 Institute of Hygiene and Epidemiology, First Faculty of Medicine, Charles University, Prague, Czech Republic; 8 Department of Epidemiology, Institute of Occupational Medicine, Lodz, Poland; 9 Core Genotyping Facility at the Advanced Technology Center of the National Cancer Institute, National Institutes of Health (NIH), Department of Health and Human Services (DHHS), Bethesda, Maryland, United States of America; Ohio State University Medical Center, United States of America

## Abstract

In the kidney vitamin D is converted to its active form. Since vitamin D exerts its activity through binding to the nuclear vitamin D receptor (*VDR*), most genetic studies have primarily focused on variation within this gene. Therefore, analysis of genetic variation in *VDR* and other vitamin D pathway genes may provide insight into the role of vitamin D in renal cell carcinoma (RCC) etiology. RCC cases (N = 777) and controls (N = 1,035) were genotyped to investigate the relationship between RCC risk and variation in eight target genes. Minimum-p-value permutation (Min-P) tests were used to identify genes associated with risk. A three single nucleotide polymorphism (SNP) sliding window was used to identify chromosomal regions with a False Discovery Rate of <10%, where subsequently, haplotype relative risks were computed in Haplostats. Min-P values showed that *VDR* (p-value = 0.02) and retinoid-X-receptor-alpha (*RXRA*) (p-value = 0.10) were associated with RCC risk. Within *VDR*, three haplotypes across two chromosomal regions of interest were identified. The first region, located within intron 2, contained two haplotypes that increased RCC risk by approximately 25%. The second region included a haplotype (rs2239179, rs12717991) across intron 4 that increased risk among participants with the *TC* (OR = 1.31, 95% CI = 1.09–1.57) haplotype compared to participants with the common haplotype, *TT*. Across *RXRA*, one haplotype located 3′ of the coding sequence (rs748964, rs3118523), increased RCC risk 35% among individuals with the variant haplotype compared to those with the most common haplotype. This study comprehensively evaluated genetic variation across eight vitamin D pathway genes in relation to RCC risk. We found increased risk associated with *VDR* and *RXRA*. Replication studies are warranted to confirm these findings.

## Introduction

Within the kidney, vitamin D is metabolized to 1,25-dihydroxycholecalciferol (1,25(OH)_2_D_3_), the active form of vitamin D [1]. The anti-carcinogenic properties of vitamin D include inhibition of clonal tumor cell proliferation, induction of immune cell differentiation and apoptosis, and decreased angiogenesis [2], [3]. Vitamin D activity is mediated through binding of 1,25(OH)_2_D_3_ to the vitamin D receptor (*VDR*), which can regulate transcription of other genes involved in cell regulation, growth, and immunity [3]–[6]. *VDR* modulates the expression of genes by forming a heterodimer complex with retinoid-X-receptors (*RXR*) [7]. This *VDR-RXR* complex is directed to the vitamin D response element (*VDRE*) in the promoter region of several 1,25(OH)_2_D_3_ regulated genes to initiate transcription [7], [8].

Since 1,25(OH)_2_D_3_ exerts its activity through nuclear *VDR*, most studies of genetic susceptibility to vitamin D related diseases have investigated variation within the *VDR* gene [4], [7], [9]–[11]. However, the concert of genes that interact with *VDR* is vast; therefore, an analysis of variation in *VDR* and other genes in the vitamin D pathway may provide insight into the role of vitamin D in renal cell carcinoma (RCC) etiology. Given the importance of vitamin D metabolism in the kidney and the lack of studies that have evaluated the relationship between vitamin D, the *VDR* gene, and RCC, we previously evaluated how three common *VDR* gene polymorphisms (*BsmI*, *FokI*, *TaqI*) modified RCC risk [12]. We observed reduced RCC risk among older subjects and among subjects with a family history of cancer who were carriers of the *f* alleles in the *FokI* single-nucleotide polymorphism (SNP) compared with subjects with the *FF* genotype [12]. However, given the limited genomic coverage of *VDR* in our previous candidate SNP investigation, and the numerous genes that interact directly with *VDR* in the vitamin D pathway, we expanded upon these findings to investigate variation in candidate genes that may interact directly with *VDR* to modify RCC risk and improved our analysis by increasing regional coverage of variation across each gene to 80–90%.

Therefore, to determine whether variation in vitamin D pathway genes modified RCC risk, we comprehensively investigated the relationship between renal cancer risk and 139 tagging SNPs across eight target genes (*VDR, RXRA, RXRB, CYP24A1, GC, STAT1, THRAP4,* and *TRAP5*) of the vitamin D pathway among cases and controls from Central Europe, an area with one of the highest rates of RCC worldwide [13]. To our knowledge, this represents one of the largest and most detailed investigations of genetic variation within the vitamin D pathway and RCC risk conducted to date.

## Results

A description of study participants and known RCC risk factors is provided in Table 1. All participants and only genotyped participants showed a similar distribution of characteristics, with the exception of sex. Cases and controls were comparable in age, but cases were more likely to have excess body mass index (BMI) (>30 kg/m^2^) and hypertension, and to have a first degree relative with cancer. The association with smoking was no longer observed after adjustment for age, BMI, hypertension, study center, and sex [14].

**Table 1:** General characteristics of participants in the CEERCC Study.

	Among All Participants					Among Genotyped Participant				
Variables	Cases		Controls			Cases		Controls		
	N	%	N	%	p-value	N	%	N	%	p-value
**Participants**	1,097	42.6	1,476	57.4		777	42.9	1,035	57.1	
**Sex**										
Males	648	59.1	952	64.5		472	60.7	648	62.6	
Females	449	40.9	524	35.5	0.01	305	39.3	387	37.4	0.42
**Age at Interview**										
<45	86	7.8	122	11.1		60	7.7	83	8.0	
45–54	278	25.3	379	34.5		197	25.4	287	27.7	
55–64	335	30.5	460	41.9		243	31.3	309	29.9	
65–74	353	32.2	452	41.2		242	31.1	318	30.7	
75+	45	4.1	63	5.7	0.50	35	4.5	38	3.7	0.30
**Mean Age (std)**	59.6 yrs. (10.3)		59.3 yrs. (10.3)			59.5 yrs. (10.4)		59.0 yrs. (10.2)		
**Center**										
Romania-Bucharest	95	8.7	160	10.8		68	8.8	94	9.1	
Poland-Lodz	99	8.7	198	13.4		80	8.7	189	18.3	
Russia-Moscow	317	28.9	463	31.4		242	31.1	313	30.2	
*Czech Republic	586	53.4	655	44.4	<0.001	387	49.8	439	42.4	<0.001
**BMI at Interview**										
<25	327	29.8	532	36.0		222	28.6	375	36.3	
25–29.9	476	43.4	620	42.0		330	42.5	432	41.9	
30+	294	26.8	324	22.0	<0.001	225	29.0	225	21.8	<0.001
**Tobacco Status**										
Never	510	46.6	599	40.7		359	46.4	420	40.7	
Ever	584	53.4	874	59.3	0.003	415	53.6	613	59.3	0.02
**Hypertension**										
No	600	54.7	906	61.4		434	55.9	638	61.7	
Yes	496	45.3	569	38.6	0.001	342	44.1	396	38.3	0.01
**Familial History of Cancer Among 1^st^ Degree Relatives**										
No	733	66.8	1,074	72.8		512	65.9	745	72.0	
Yes	364	33.2	402	27.2	0.001	265	34.1	290	28.0	0.01

* Brno, Olomouc, Prague, Ceske-Budejovice.

Table 2 lists results for global gene-based tests of association with case/control status using the minimum p-value permutation (Min-P) test. *VDR* (p-value = 0.02) and *RXR-alpha (RXRA)* (p-value = 0.10) genes were most strongly associated with RCC risk. Both the minimum p-value for all tagging SNPs within the gene and minimum false discover rate (FDR) adjusted p-values for the three SNP haplowalk sliding window for *VDR* and *RXRA* were significantly associated with RCC risk.

**Table 2:** Vitamin D pathway gene-based global, trend, and haplowalk minimum p-values for associations with renal cancer risk.

Target Gene Name (*Alias*)	Target Gene Function	Chromosome Location	Number of Tag SNPs	Adjusted Min-P Test	*Minimum Adjusted p-trend	†Haplowalk
***VDR***: Vitamin D 1,25 dihydroxyvitamin D_3_ Receptor (*NR111*)	Encodes the nuclear hormone receptor for vitamin D_3_ and is principally involved in mineral metabolism through the receptor; regulates variety of other metabolic pathways.	12q13.11	29	0.024	0.002	0.045
***RXRA***: Retinoid X Receptor, alpha (*FLJ16020, FLJ16733, MGC102720, NR2B1*)	Mediates the biological effects of retinoids; as homo- or heterodimers, binds to the vitamin D receptor gene and regulates transcription.	9q34.3	18	0.100	0.011	0.007
***STAT1***: Signal Transducer and activator of Transcription 1, 91kDa (*DKFZp686B04100, ISGF-3, STAT91*)	Phosphorylated by receptor associated kinases; protein forms homo- or heterodimers that translocate to the cell nucleus where they act as transcription activators.	2q32.2	21	0.138	0.022	0.085
***RXRB***: Retinoid X Receptor, beta (*DAQB-314F24.5, DAUDI6, H-2RIIBP, MGC1831, NR2B2, RCoR-1*)	Forms homodimers with the retinoic acid, thyroid hormone, and vitamin D receptors, increasing binding and transcription on response elements.	6p21.3	8	0.202	0.093	0.262
***GC***: Group-Specific Component Vitamin D Binding Protein (*DBP*)	Binds vitamin D and its plasma metabolites, transporting them to target tissues.	4q12–q13	11	0.246	0.037	0.090
***THRAP5***: Thyroid Hormone Receptor Associated Protein 5 (*DRIP92, MED16, TRAP95*)	Selectively interacts with vitamin D receptors, mediating the action of vitamin D by binding and controlling the transcription of hormone-sensitive genes.	19p13.3	11	0.384	0.113	0.192
***CYP24A1***: Cytochrome P450, Family 2, Subfamily A, Polypeptide 1 (*CP24, CYP24, MGC126273, MGC126274, P450-CC24*)	Initiates degradation of 1,25-dihydroxyvitamin D_3_; regulates levels of vitamin D3; plays a role in calcium homeostasis and vitamin D endocrine system.	20q13	35	0.630	0.035	0.729
***THRAP4***: Thyroid Hormone Receptor Associated Protein 4 (*ARC100, CRSP100, CRSP4, DRIP100, KIAA0130, MGC8748, MED24, TRAP100*)	Induces gene expression and selectively interacts with vitamin D receptors mediating the action of vitamin D by binding and controlling the transcription of hormone-sensitive genes.	17q21.1	6	0.936	0.580	0.927

* Adjusted minimum p-value for all tagging SNPs in a targeted gene using additive model.

† Minimum FDR adjusted p-value for 3- SNP haplowalk sliding window analysis.

*† Adjusted for age, sex, study center, and smoking status (ever, never).

**Figure 1:**
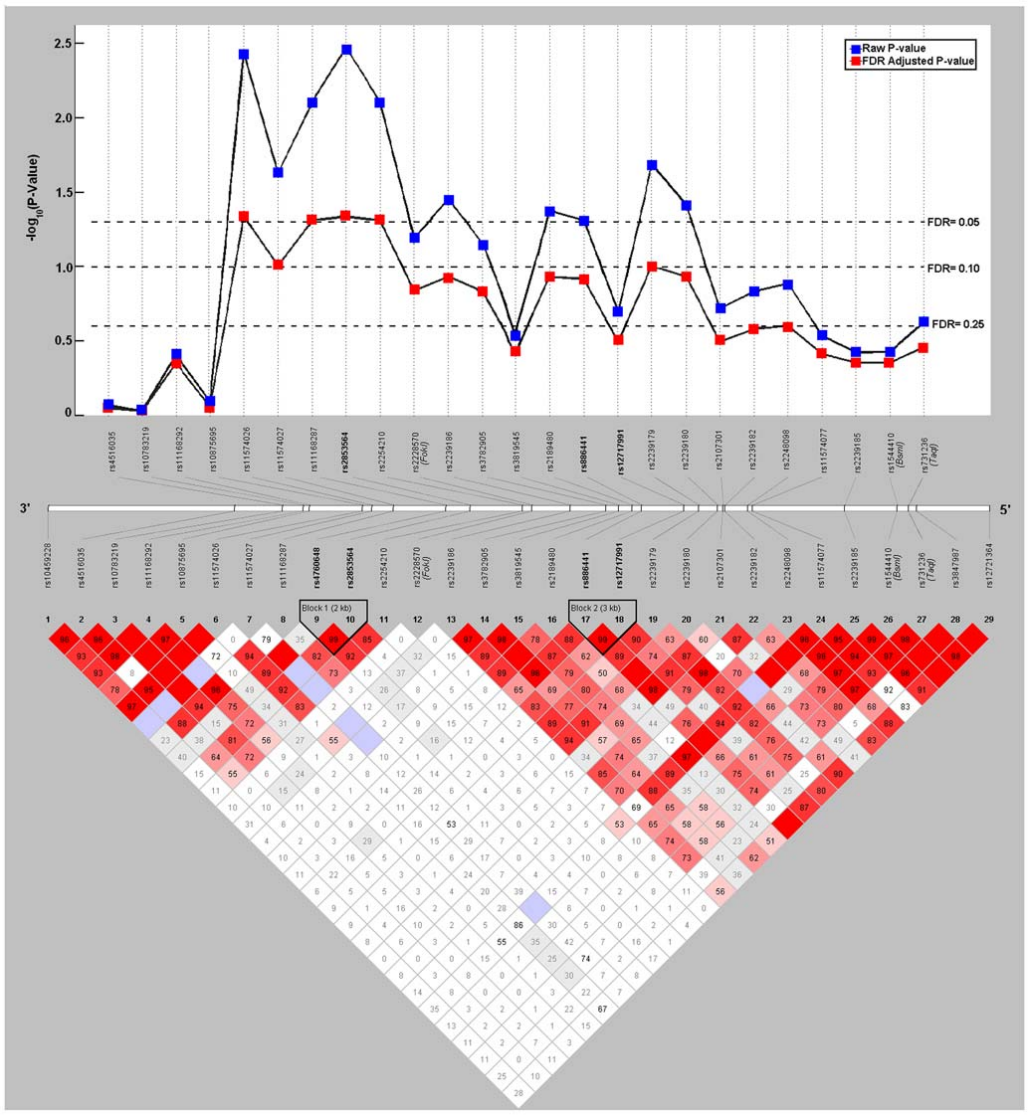
VDR HaploWalk and Haploview analysis. Top: HaploWalk analysis identified two chromosomal regions of interest in the vitamin D receptor (VDR) gene that were significant at an FDR of 10%. Region one included SNPs 3′- rs4760648, rs2853564, rs2254210 -5′; region two included SNPs 3′- rs12717991, rs2239179 -5′. Bottom: Haploview analysis also identified two haplotype blocks within the same chromosomal regions of interest.

**Figure 2:**
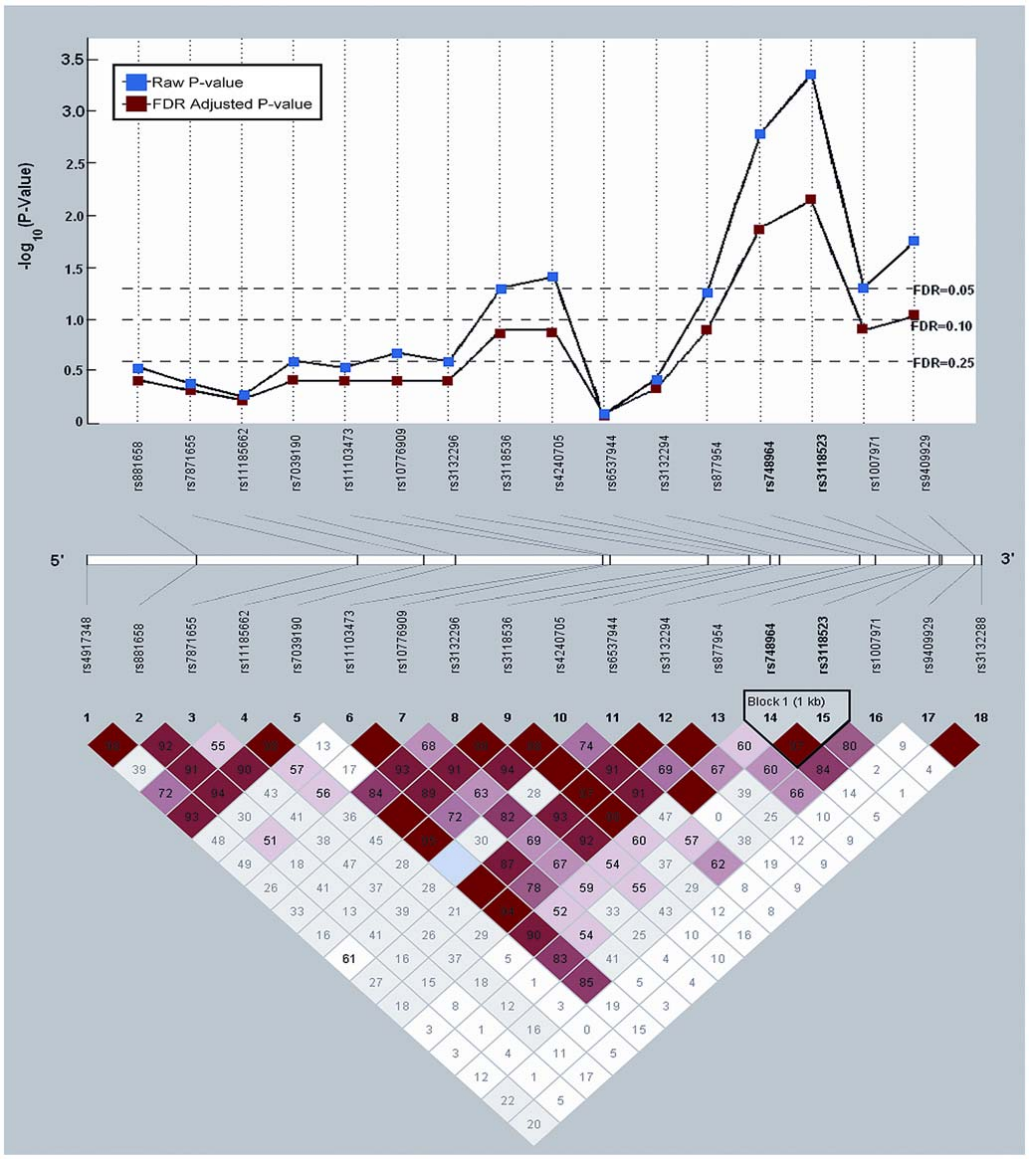
RXRA HaploWalk and Haploview analysis. Top: HaploWalk analysis identified a single chromosomal region of interest in the retinoid-X-receptor-alpha (RXRA) gene that was significant at an FDR of 10%. This region included SNPs 5′- rs748964, rs3118523 -3′. Bottom: Haploview analysis also identified one haplotype block within the same chromosomal region of interest.

Subsequently, HaploWalk based methods were used to identify regions of interest in each gene that remained significant at an FDR of less than 10%. Three significant *VDR* haplotypes (Table 3 ) were identified in two regions that were associated with increased RCC risk. The first region (Figure 1), located within intron 2, showed subjects with the *AGC* (OR = 1.29; 95% CI = 1.10–1.52; p-value = 0.002) or *GAC* (OR = 1.25; 95% CI = 1.04–1.51; p-value = 0.02) haplotype were at a significantly increased risk compared to patients with the most common referent haplotype, *GAT*. Increased risk appeared to be driven by the *T* to *C* change at the third loci. The second region (Figure 1), centered around intron 4, also increased RCC risk among participants with the *TC* (OR = 1.31; 95% CI = 1.09–1.57; p-value = 0.004) haplotype compared to participants with most common referent haplotype, *TT*. The R adjusted global p-values for both of these regions were 0.04 (Table 3 ). Across the *RXRA* gene (Figure 2 ), a single region located downstream, 3′ of the coding sequence, was shown to be associated with increased RCC risk among individuals with the *CG* (OR = 1.35; 95% CI = 1.11–1.66; p-value = 0.003) haplotype compared to subjects with the most common haplotype, *GA*. The R adjusted global p-value for this region was 0.03.

**Table 3:** Renal cancer risk and haplotypes in genes in the vitamin D pathway.

Haplotypes	Cases (%)	Controls (%)	OR	(LCI-UCI)	P-value	Global
† ***VDR***						
Region 1						
**5′-rs2254210, rs2853564, rs4760648-3′**						
**G-A-T**	37.8	42.7	1.00			
**A-G-C**	32.3	28.9	1.29	(1.10–1.52)	**0.002**	
**G-A-C**	20.3	18.4	1.25	(1.04–1.51)	**0.02**	
**G-G-C**	6.5	7.1	1.04	(0.78–1.38)	0.78	
						**0.04**
Region 2						
**5′-rs2239179, rs12717991-3′**						
**T-T**	37.9	41.6	1.00			
**C-C**	37.0	37.1	1.07	(0.92–1.25)	0.36	
**T-C**	23.6	19.7	1.31	(1.09–1.57)	**0.004**	
						**0.04**
‡ ***RXRA***						
Region 1						
**5′-rs748964, rs3118523-3′**						
**G-A**	78.9	82.8	1.00			
**G-G**	6.5	5.9	1.11	(0.85–1.46)	0.44	
**C-G**	14.3	11.1	1.35	(1.11–1.66)	**0.003**	
						**0.03**

Adjusted for age (continuous), sex, study center, and smoking habit (ever, never).

† *VDR* chr12 region1: 46466932-46559981; region 2: 46545393-46544033.

‡ *RXRA* chr9 region1: 136475342-136473910.

Results for individual analyses of these SNPs are provided in Supplemental Table S1. Thirteen SNPs in five vitamin D pathway genes were significantly associated with RCC risk using an additive model (number of significant tag SNPs): *VDR*(5), *RXRA*(5), *CYP24A1*(1), *GC*(1), and *STAT1*(1). No statistically significant interactions between these SNPs and potential confounders (i.e. BMI, age, sex, smoking status, hypertension, and a family history of cancer) were detected (data not shown). No difference in association between individual SNPs and RCC risk was observed when analyses were restricted to RCC cases histologically diagnosed with clear cell subtype (data not shown) nor when analyses were restricted to cases with only high grades tumors (data not shown).

## Discussion

In this analysis of 139 tagging SNPs across eight vitamin D pathway genes, we identified three regions within two genes that were significantly associated with RCC risk. Both genes, *VDR* and *RXRA*, for which we hypothesized *a priori* would be associated with risk, contained regions with significant associations. Across the *VDR* gene, three haplotypes within two regions (intron 2 and intron 4) were significantly associated with increased risk. Across the *RXRA* gene, RCC risk was higher among those with one particular haplotype located 3′ of the coding region.

Only a handful of studies have comprehensively analyzed genetic variation in the *VDR* gene in relation to cancer [15], [16], [17]. A case-control study of 630 incident prostate cancer patients was conducted to comprehensively analyze genetic variation in the *VDR* gene; twenty-two SNPs were genotyped to capture a high percentage of variation in the *VDR* gene [17]. A two-fold increase in prostate cancer risk was observed for two *VDR* loci (rs2107301 and rs2238135), which were located within introns 2 and 4, respectively [17]. Though information is limited regarding the association between *VDR* variants and RCC risk, genetic susceptibility studies for common *VDR* polymorphisms, such as *BsmI*, *FokI*, *TaqI, and ApaI*, have been shown to modify RCC risk [15], [16]. A recent case-control study of 135 RCC patients found that participants with the *AA* genotype at the *ApaI* site had a significant increase in RCC risk (OR = 2.59; p-value = 0.01); cases with the *AA* genotype also were found to have lower survival rates (Relative Risk = 3.3; p-value = 0.04) compared to cases with the *Aa* or *aa* genotypes [16]. A two-fold increase in cancer risk was also reported among participants with the *TT* genotype at the *TaqI* site in a second case-control study of 102 RCC patients compared to subjects with the *Tt* or *tt* genotype (p-value = 0.001) [15].

Similarly, few previous studies of RCC risk and variation across vitamin D pathway genes have been conducted. A small number of polymorphisms in the *VDR* gene, such as *poly(A)*, *TaqI*, and *BsmI*, have been speculated to result in variation of *VDR* expression and are hypothesized to subsequently result in changes to circulating levels of active vitamin D [15], [18], [19]. Altered *VDR* protein expression has been reported in a number of tumor types including breast, malignant gliomas, prostate, and colon cancer [20]. Moreover, previous epidemiological studies have reported that increased binding of vitamin D to *VDR* is associated with decreased RCC risk, and that active levels of vitamin D_3_ in serum are significantly lower in RCC patients compared to population controls [15], [16], [19]. Together these results indicate that vitamin D levels influence RCC risk. While results have been mixed concerning associations between variants in the *RXR* gene and cancer risk [21]–[24], *in vitro* animal and human studies have shown that elevated levels of *RXRA* increases the antiproliferative effects of 1,25(OH)_2_D_3_ [25], [26].

*In vitro* and animal studies suggest that vitamin D and its metabolites may impede carcinogenesis by stimulating cell differentiation, inhibiting cell proliferation typically characterized by G0/G1 cell cycle arrest, apoptosis, and suppression of invassion, angiogenesis, and metastasis [3]–[6]. In laboratory studies, 1,25 hydroxyvitamin D has been shown to significantly reduce tumor growth for a variety of cancers including colon, breast, prostate and lung [18]. The use of vitamin D or its analogs as chemopreventive agents in humans, although limited, have shown beneficial effects. In a clinical trial for patients with inoperable advanced hepatocellular carcinoma a daily dose of 10 mg of Seocalcitol (a vitamin D analog) for up to one year resulted in a reduction in tumor dimension [27]. Additionally, among 37 patients with metastatic androgen-independent prostate cancer, who were treated with a high dose treatment of oral calcitriol/docetaxel (active form of vitamin D/mitotic inhibitor), prostate-specific antigen reductions of at least 50% was observed [28].

Although the magnitude of association between RCC risk and *VDR* and *RXRA* gene variants in our study may be small, some of these variants are relatively common in the population and therefore may be associated with a much higher attributable risk as a whole compared to rare high penetrance genes. Furthermore, the *VDR* variants associated with RCC risk in this study were intronic and were not located near intron-exon boundaries that may produce splicing errors. These alleles seem to have no effect on the expression levels or activity of translated *VDR* protein and it is hypothesized that they are correlated with other variants with functional relevance. Synonymous SNPs are often disregarded in many genetic susceptibility studies based on the assumption that they do not alter amino acid sequence, which would subsequently affect protein structure and function, nor protein expression [29]. Yet, the list of synonymous mutations associated with human diseases is growing [29], [30]. Recent genetic studies have shown that synonymous mutations are not random and may play a significant role in disease etiology since synonymous SNPs can affect protein expression and function by altering mRNA stability [29], [31], [32]. Studies solely focusing on genetic variations within exonic regions of DNA that lead to changes in protein sequence may be simplistic and may not represent the larger picture that is evolving between genetic variation and disease [33].

In addition to being one of the first genetic susceptibility studies to comprehensively examine the association between *VDR* and seven other vitamin D pathway genes in relation to RCC risk, an additional strength of our study includes the use of HapMap data to tag genes of interest using high (80–90%) genomic coverage by tagging genomic regions, both upstream (20 kb) and downstream (10 kb) to reduce chance of false negative findings. In this study, we also observed high participation rates, used newly diagnosed cases, included only histologically confirmed cancers, and collected biologic materials from a high proportion of subjects. The large sample size of this study provided sufficient statistical power to detect relatively small associations between genotypes and risk. Analysis of genes with significant global p-values reduced the risk of Type I errors in our study, while the use of two different Haplotype-based methods (HaploWalk and HaploStats) reduced the possibility of finding significant results based on chance. Furthermore, while hospital-based case-control studies have potential limitations due to the lack of population controls, these studies can improve response rates for the intense collection of biological specimens and therefore reduce the chances of bias in the assessment of gene-environment interactions [34]. Lastly, we did not employ a direct marker for vitamin D among these study subjects. While there is general agreement that serum 25(OH)D level is the best indicator of current vitamin D status, the biochemical marker has a short half-life and would not necessarily reflect long-term vitamin D status, particularly for case-control studies where cases have just recently been diagnosed [35].

In conclusion, our *a priori* hypothesis was supported and we observed that genetic variations in *VDR* and *RXRA* were significantly associated with RCC risk. However, since our results are observed within introns rather than exons and because we are only beginning to understand the role of genetic variation in intronic regions and their effect on disease development, additional studies of genetic variations of these two genes and RCC risk are warranted.

## Materials and Methods

### Study Subjects

Details of the Central and Eastern European Renal Cell Carcinoma (CEERCC) study have been previously reported [36]. Briefly, the CEERCC study is a hospital-based case-control study of renal cell cancer conducted between 1999 and 2003 in seven centers in four Central and Eastern European countries (Moscow, Russia; Bucharest, Romania; Lodz, Poland; and Prague, Olomouc, Ceske-Budejovice, and Brno, Czech Republic). Each center followed an identical protocol and was responsible for recruiting a consecutive group of newly diagnosed cases of kidney cancer as well as a comparable group of hospital controls. Controls in all centers were chosen among subjects admitted as in- or out-patients in the same hospital as the cases, with conditions unrelated to tobacco or genitourinary disorders. No single disease made up more than 20% of the control group. All subjects were residents of the study areas for at least one year at the time of recruitment and were of Caucasian descent.

Cases between 20 and 79 years of age were ascertained through a rapid reporting system. Participating physician and other professional staff members visiting the urology, surgery, radiology, or pathology departments of participating hospitals regularly identified patients admitted for kidney cancer work-up. All patients suspected of having kidney cancer were approached for interview, but only histologically confirmed cases were included in the final analysis. All tumors were centrally reviewed to confirm diagnosis and histology. The majority of renal tumors were clear cell carcinomas (83.4%), while other subtypes included papillary (7%), chromophobe (2.4%), oncocytoma (2.3%), oncocytic neoplasms (0.2%), transitional cell carcinomas (1.1%), and unclassified (3.6%). Controls were frequency-matched to cases on age (±3 years), sex, and study center. A total of 1,097 confirmed incident RCC cases and 1,476 controls were included in this study. Genomic DNA was obtained from 987 of 1,097 (90%) of cases and 1,298 of 1,476 (88%) of controls. The response rates across study centers among eligible subjects who were requested to participate ranged from 90.0–98.6% for cases and 90.3–96.1% for controls.

### Interview

Cases and controls were interviewed with the same questionnaire [37]. Information on demographic characteristics, medical histories, and lifestyle factors was obtained through in-person interviews by trained personnel using standardized lifestyle and food frequency questionnaires. Cases were interviewed within three months of diagnosis. Written consent for participation was obtained from all study subjects, and ethical approval was obtained for all study centers.

### Laboratory Procedures

Genomic DNA was extracted from whole blood buffy coat using a standard phenol chloroform method. Genotyping was conducted with a GoldenGate® Oligo Pool All (OPA) assay by Illumina® (www.illumina.com) at the National Cancer Institute's (NCI's) Core Genotyping Facility (CGF) where lab staff was blinded to cases/control status. Suitable quantity and quality of genomic DNA was obtained from a subset of 777 (70.8%) RCC cases and 1,035 (70.1%) controls, due to strict quality and quantity criteria for analysis using the Illumina® OPA panel.

A literature review of vitamin D associated genes helped identify eight candidate genes (*VDR, RXRA, RXRB, CYP24A1, GC, STAT1, THRAP4* and *THRAP5*) that were ultimately selected for their involvement in vitamin D metabolism, transport, binding, function and/or expression, which may affect the mechanisms through which vitamin D may influence cancer risk. Since it is well known that *VDR* elicits a transcriptional response by forming a heterodimer complex with the retinoid-X-receptor, in this study both *VDR* and *RXR* were the primary genes suspected *a priori* to modify renal cancer risk. The group specific component *(GC)* vitamin D binding protein was selected for its involvement as the major carrier of vitamin D in plasma [38]. Cytochrome P450, family 24, subfamily A, polypeptide 1 (*CYP24A1*) was selected for its involvement in degrading and regulating 1,25(OH)_2_D_3_ levels [39], [40]. Lastly, the signal transducer and activator of transcription 1 (*STAT1*) and the thyroid hormone receptor associated protein 4 and 5 (*THRAP4* and *THRAP5*) genes were selected since studies indicate that these genes may induce or suppress transcription by interacting with the *VDR-RXR* complex [41], [42].

Tag SNPs were selected to provide 80–90% genomic coverage across the genomic regions of interest. To ensure thorough coverage of the targeted region, additional coverage of regions both upstream (20 kb) of the start of transcription and downstream (10 kb) of the last exon using HapMap CEU data (http://www.hapmap.org) among SNPs with minor allele frequencies of at least 5% and an r^2^≥80% were selected. Additionally, non-synonymous SNPs or those correlated with polymorphisms with potential functional significance were included in analysis. In total, 139 SNPs in eight vitamin D-related pathway target genes were selected for analysis. The total length of sequence analyzed across all genes was 495,664 bases (*VDR*: 37,421 bases, *RXRA*: 144,022 bases, *RXRB*: 36,257 bases, *CYP24A1*: 50,537 bases, *GC*: 72,479 bases, *STAT1*: 75,061 bases, *THRAP4*: 39,862 bases, and *THRAP5*: 40,025 bases). Replicate quality control samples (5% samples) were interspersed among genotyping plates. The genotyping failure rate for the selected SNPs was 4.8% (7/146). The concordance rate between duplicate DNA samples ranged from 93–100% and completion rates ranged from 98–100%. The genotype frequencies among controls did not differ from the expected Hardy-Weinberg equilibrium proportions (p>0.05). All SNPs and assay information are reported in NCI's SNP500Cancer Database (http://snp500cancer.nci.nih.gov) [43].

### Statistical Analysis

The distributions of selected characteristics and known RCC risk factors (sex, age, smoking habits, hypertension, BMI, family history of cancer, and country of residence) were compared between genotyped cases and controls using the Chi-square test. These characteristics were further evaluated to determine if associations between SNPs and RCC were modified; however, no significant interactions were observed between cancer risk, RCC risk factors, and SNPs (data not shown).

The associations between individual SNPs and RCC risk were estimated by calculating odds ratios (ORs) and 95% confidence intervals (95% CI) using unconditional logistic regression. Interactions were tested comparing regression models with and without interaction terms using a likelihood ratio test (LRT). All regression models were adjusted for age (continuous), sex, study center, and smoking habit (ever, never). Additional risk factors (i.e. hypertension, BMI, and family history of cancer) were also considered, however, these variables did not affect risk estimates by at least 10%. To avoid redundant analyses, only one SNP was evaluated when high correlations were observed between two SNPs (r^2^>0.85). Of the 139 SNPs genotyped, two pairs of SNPs in the *VDR* gene were found to be highly correlated (r^2^ = 0.95 for rs1544410 (*BsmI*) and rs731236 (*TaqI*); r^2^ = 0.89 for rs2248098 and rs2239185) and therefore only one SNP was used per pair (rs1544410 (*BsmI*) and rs2248098).

Since the most common form of renal cell carcinoma is of clear cell type, associations between individual SNPs and RCC risk were also evaluated among cases histologically diagnosed with this subtype of renal cancer. However restricting cases to those with clear cell type did not affect risk estimates by at least 10% (data not shown). Similarly, analyses restricted to cases with only high grades tumors did not affect risk estimates (data not shown).

Risk estimates were calculated for the heterozygous and homozygous variant genotypes relative to the common homozygous genotype assuming an additive model. When the frequency of the homozygous variant allele was less than 5% among controls, a dominant model was used to determine risk estimates for the presence and absence of the variant allele. Associations between SNP variants and RCC were assessed using an additive model (i.e. linear test of trend for the number of copies of the variant allele (0, 1, 2)) or a dominant model (i.e. Wald chi-square test for the presence or absence of the variant allele (0, 1)). To assess global significance of association with genes, a minimum p-value permutation (Min-P) test was used for analysis because it corrects for multiple testing while also accounting for correlations between SNPs within a gene [44]. Genes that had a significant Min-P test at a cut-off level of 10% were selected for further analysis. Analysis for genes that did not meet this criterion is available in Supplemental Figure S1, S2, S3, S4, S5, S6.

HaploWalk, a haplotype-based sliding window analytic method, was used to evaluate candidate genes with N = *K* SNPs. The HaploWalk procedure considered a three SNP window for each SNP from SNP *2* through SNP *K-1*. For each window, haplotype frequencies in cases and controls were reconstructed using the EM algorithm [45], and a Wald test was used to screen for association with case-control status [44]. In the initial screening phase, no adjustment was made for potential confounders. The Wald test used a threshold value of 5%, such that haplotypes in cases and controls that had an estimated frequency below the threshold in controls were pooled into an ‘other’ rare haplotype category for testing. To account for multiple testing across the *K* SNPs, the *K-2* p-values (one for each window) were adjusted for multiple comparisons using the False Discovery Rate (FDR)-controlling procedure of Benjamini and Hochberg [46]. Haplotype windows with an FDR-adjusted value of <10% were promoted to the second stage.

In stage two, haplotype blocks identified with HaploWalk were analyzed in relation to RCC risk using Haplostats (R version 2.4.0; http://www.r-project.org) [47], adjusted for age, sex, study center, and smoking habit. Additionally, we used standard methods to identify haplotypes using the Haploview program version 3.32 [48]. Linkage disequilibrium between markers was assessed by calculating pairwise Lewontin's D' and r^2^ using Haploview among population controls [48]. Heterogeneity of genotype frequencies among countries was evaluated by using the likelihood ratio test to compare the fit of models with and without interaction terms, but we did not find evidence of heterogeneity across populations. No evidence of population stratification was apparent from a principal components analysis of a genome wide association study conducted in this population [49], and the likelihood of this is small among European populations [50]. Associations between common haplotypes (>5% frequency) and RCC risk were evaluated by computing ORs and 95% CIs using the most common haplotype category for comparison. All analyses were conducted in STATA 9.0 unless otherwise specified (STATA Corporation, College Station, TX).

## Supporting Information

Table S1 SNP-based analysis main effects for vitamin D pathway genes
(1.73 MB DOC)

Figure S1 RXRB HaploWalk and Haploview analysis. Top: HaploWalk analysis identified no chromosomal regions of interest in the retinoid-X-receptor-beta (RXRB) gene that were significant at an FDR of 10%. The Min-P test for this gene was not statistically significant (p-value = 0.20). Bottom: Haploview analysis identified two haplotype blocks; none were associated with RCC risk when analyzed in Haplostats.
(0.24 MB TIF)

Figure S2 CYP24A1 HaploWalk and Haploview analysis. Top: HaploWalk analysis identified no chromosomal regions of interest in the cytochrome P450, family 24, subfamily A, polypeptide 1 (CYP24A1) gene that were significant at an FDR of 10%. The Min-P test for this gene was not statistically significant (p-value = 0.63). Bottom: Haploview analysis identified several haplotype blocks; none were associated with RCC risk when analyzed in Haplostats.
(0.76 MB TIF)

Figure S3 GC HaploWalk and Haploview analysis. Top: HaploWalk analysis identified no chromosomal regions of interest in the group specific component (GC) vitamin D binding protein that were significant at an FDR of 10%. The Min-P test for this gene was not statistically significant (p-value = 0.25). Bottom: Haploview analysis identified one haplotype block which was not associated with RCC risk when analyzed in Haplostats.
(2.86 MB TIF)

Figure S4 STAT1 HaploWalk and Haploview analysis. Top: HaploWalk analysis identified a single chromosomal region of interest in the signal transducer and activator of transcription 1 (STAT1) gene that was significant at an FDR of 10%. This region included SNPs 3′- rs3755821, rs6751855, rs1467199, rs13029532 -5′. The Min-P test for this gene was not statistically significant (p-value = 0.14). Bottom: Haploview analysis also identified one haplotype block within the same chromosomal region of interest. Haplostats analysis adjusted for age, sex, study center, and smoking habit showed that subjects with the CGGA haplotype had an increased risk of RCC (OR = 1.21; 95% CI = 1.01–1.45; p-value = 0.04) compared to subjects with the referent haplotype, GACA.
(1.76 MB TIF)

Figure S5 THRAP4 HaploWalk and Haploview analysis. Top: HaploWalk analysis identified no chromosomal regions of interest in the thyroid hormone receptor associated protein 4 (THRAP4) gene that were significant at an FDR of 10%. The Min-P test for this gene was not statistically significant (p-value = 0.94). Bottom: Haploview analysis identified one haplotype block which was not associated with RCC risk when analyzed in Haplostats.
(1.42 MB TIF)

Figure S6 THRAP5 HaploWalk and Haploview analysis. Top: HaploWalk analysis identified no chromosomal regions of interest in the thyroid hormone receptor associated protein 5 (THRAP5) gene that were significant at an FDR of 10%. The Min-P test for this gene was not statistically significant (p-value = 0.38). Bottom: Haploview analysis identified several haplotype blocks; none were associated with RCC risk when analyzed in Haplostats.
(0.64 MB TIF)
