# Supplementary material for: Analysis of SNPs and Haplotypes in Vitamin D Pathway Genes and Renal Cancer Risk
Source: PLoS One. 2009 Sep 15;4(9):e7013. doi: 10.1371/journal.pone.0007013 (PMC2737618; doi:10.1371/journal.pone.0007013)
Supplement: Table S1 — SNP-based analysis main effects for vitamin D pathway genes (1.73 MB DOC) [file pone.0007013.s001.doc]

| **Table S1. SNP-based analysis main effects for vitamin D pathway genes** | | | | | | | | | | | | | |
| --- | --- | --- | --- | --- | --- | --- | --- | --- | --- | --- | --- | --- | --- |
|  |  |  |  |  |  |  |  |  |  |  |  |  |  |
|  |  | **Cases** | |  | **Controls** | |  |  |  |  |  |  |  |
|  |  | **N** | **%** |  | **N** | **%** |  | **OR** | **LCI** | **-** | **UCI** | **p-trend** | |
|  |  |  |  |  |  |  |  |  |  |  |  |  |  |
| **rs1555439** | **(-15343G>T)** |  |  |  |  |  |  |  |  |  |  |  |  |
| (PFDN4-01) | GG | 508 | 65.5 |  | 679 | 65.9 |  | 1.00 |  |  |  |  |  |
|  | GT/TT | 268 | 34.5 |  | 352 | 34.1 |  | 1.04 | 0.85 | **-** | 1.27 |  |  |
|  |  |  |  |  |  |  |  |  |  |  |  |  |  |
|  |  |  |  |  |  |  |  |  |  |  |  |  | 0.75 |
| **rs2585421** | **(-16904T>C)** |  |  |  |  |  |  |  |  |  |  |  |  |
| (CYP24A1-54) | TT | 550 | 70.8 |  | 774 | 74.8 |  | 1.00 |  |  |  |  |  |
|  | CT/CC | 227 | 29.2 |  | 261 | 25.2 |  | 1.18 | 0.95 | **-** | 1.46 |  |  |
|  |  |  |  |  |  |  |  |  |  |  |  |  |  |
|  |  |  |  |  |  |  |  |  |  |  |  |  | 0.17 |
| **rs2252928** | **(-16551T>A)** |  |  |  |  |  |  |  |  |  |  |  |  |
| (CYP24A1-85) | TT | 609 | 78.4 |  | 816 | 79.1 |  | 1.00 |  |  |  |  |  |
|  | AT/AA | 168 | 21.6 |  | 215 | 20.9 |  | 1.09 | 0.86 | **-** | 1.37 |  |  |
|  |  |  |  |  |  |  |  |  |  |  |  |  |  |
|  |  |  |  |  |  |  |  |  |  |  |  |  | 0.36 |
| **rs6023012** | **(-16454G>A)** |  |  |  |  |  |  |  |  |  |  |  |  |
| (CYP24A1-83) | GG | 297 | 38.6 |  | 414 | 40.3 |  | 1.00 |  |  |  |  |  |
|  | AG | 361 | 46.9 |  | 487 | 47.4 |  | 1.01 | 0.82 | **-** | 1.24 |  |  |
|  | AA | 111 | 14.4 |  | 127 | 12.4 |  | 1.20 | 0.89 | **-** | 1.62 |  |  |
|  |  |  |  |  |  |  |  |  |  |  |  |  | 0.33 |
| **rs765058** | **(-14373G>A)** |  |  |  |  |  |  |  |  |  |  |  |  |
| (CYP24A1-84) | GG | 411 | 53.3 |  | 571 | 55.3 |  | 1.00 |  |  |  |  |  |
|  | AG | 294 | 38.1 |  | 396 | 38.4 |  | 1.01 | 0.83 | **-** | 1.24 |  |  |
|  | AA | 66 | 8.6 |  | 65 | 6.3 |  | 1.47 | 1.01 | **-** | 2.14 |  |  |
|  |  |  |  |  |  |  |  |  |  |  |  |  | 0.15 |
| **rs2585424** | **(-8460G>T)** |  |  |  |  |  |  |  |  |  |  |  |  |
| (CYP24A1-86) | GG | 640 | 82.7 |  | 894 | 86.4 |  | 1.00 |  |  |  |  |  |
|  | GT/TT | 134 | 17.3 |  | 141 | 13.6 |  | 1.34 | 1.03 | **-** | 1.74 |  |  |
|  |  |  |  |  |  |  |  |  |  |  |  |  |  |
|  |  |  |  |  |  |  |  |  |  |  |  |  | 0.07 |
| **rs2208588** | **(-6852A>T)** |  |  |  |  |  |  |  |  |  |  |  |  |
| (CYP24A1-81) | AA | 228 | 29.3 |  | 334 | 32.3 |  | 1.00 |  |  |  |  |  |
|  | AT | 381 | 49.0 |  | 500 | 48.3 |  | 1.09 | 0.87 | **-** | 1.35 |  |  |
|  | TT | 168 | 21.6 |  | 201 | 19.4 |  | 1.22 | 0.93 | **-** | 1.60 |  |  |
|  |  |  |  |  |  |  |  |  |  |  |  |  | 0.16 |
| **rs2426498** | **(-6562C>G)** |  |  |  |  |  |  |  |  |  |  |  |  |
| (CYP24A1-52) | CC | 598 | 77.0 |  | 807 | 78.0 |  | 1.00 |  |  |  |  |  |
|  | CG/GG | 179 | 23.0 |  | 228 | 22.0 |  | 1.02 | 0.81 | **-** | 1.29 |  |  |
|  |  |  |  |  |  |  |  |  |  |  |  |  |  |
|  |  |  |  |  |  |  |  |  |  |  |  |  | 0.89 |
| **rs2248359** | **(-1399C>T)** |  |  |  |  |  |  |  |  |  |  |  |  |
| (CYP24A1-01) | CC | 268 | 34.5 |  | 404 | 39.1 |  | 1.00 |  |  |  |  |  |
|  | CT | 374 | 48.2 |  | 473 | 45.7 |  | 1.19 | 0.96 | - | 1.46 |  |  |
|  | TT | 134 | 17.3 |  | 157 | 15.2 |  | 1.33 | 1.00 | - | 1.76 |  |  |
|  |  |  |  |  |  |  |  |  |  |  |  |  | **0.03** |
| **rs6022999** | **(IVS3+103A>G)** |  |  |  |  |  |  |  |  |  |  |  |  |
| (CYP24A1-82) | AA | 472 | 60.8 |  | 651 | 63.0 |  | 1.00 |  |  |  |  |  |
|  | AG/GG | 304 | 39.2 |  | 383 | 37.0 |  | 1.14 | 0.93 | **-** | 1.39 |  |  |
|  |  |  |  |  |  |  |  |  |  |  |  |  |  |
|  |  |  |  |  |  |  |  |  |  |  |  |  | 0.21 |
| **rs13038432** | **(IVS3+814A>G)** |  |  |  |  |  |  |  |  |  |  |  |  |
| (CYP24A1-50) | AA | 680 | 89.6 |  | 886 | 86.9 |  | 1.00 |  |  |  |  |  |
|  | AG/GG | 79 | 10.4 |  | 133 | 13.1 |  | 0.81 | 0.60 | **-** | 1.10 |  |  |
|  |  |  |  |  |  |  |  |  |  |  |  |  |  |
|  |  |  |  |  |  |  |  |  |  |  |  |  | 0.23 |
| **rs4809960** | **(IVS4+58T>C)** |  |  |  |  |  |  |  |  |  |  |  |  |
| (CYP24A1-67) | TT | 422 | 54.4 |  | 605 | 58.6 |  | 1.00 |  |  |  |  |  |
|  | CT | 305 | 39.3 |  | 360 | 34.8 |  | 1.18 | 0.96 | **-** | 1.44 |  |  |
|  | CC | 49 | 6.3 |  | 68 | 6.6 |  | 1.00 | 0.67 | **-** | 1.49 |  |  |
|  |  |  |  |  |  |  |  |  |  |  |  |  | 0.31 |
| **rs4809959** | **(IVS4+272G>A)** |  |  |  |  |  |  |  |  |  |  |  |  |
| (CYP24A1-79) | GG | 238 | 30.6 |  | 277 | 26.8 |  | 1.00 |  |  |  |  |  |
|  | AG | 369 | 47.5 |  | 511 | 49.5 |  | 0.84 | 0.67 | **-** | 1.05 |  |  |
|  | AA | 170 | 21.9 |  | 245 | 23.7 |  | 0.80 | 0.61 | **-** | 1.05 |  |  |
|  |  |  |  |  |  |  |  |  |  |  |  |  | 0.09 |
| **rs2181874** | **(IVS4+1653G>A)** |  |  |  |  |  |  |  |  |  |  |  |  |
| (CYP24A1-07) | GG | 450 | 58.0 |  | 619 | 60.0 |  | 1.00 |  |  |  |  |  |
|  | AG | 274 | 35.3 |  | 351 | 34.0 |  | 1.08 | 0.88 | **-** | 1.32 |  |  |
|  | AA | 52 | 6.7 |  | 61 | 5.9 |  | 1.19 | 0.80 | **-** | 1.77 |  |  |
|  |  |  |  |  |  |  |  |  |  |  |  |  | 0.32 |
| **rs2762941** | **(IVS4-1280G>A)** |  |  |  |  |  |  |  |  |  |  |  |  |
| (CYP24A1-63) | GG | 344 | 44.7 |  | 462 | 44.9 |  | 1.00 |  |  |  |  |  |
|  | AG | 339 | 44.1 |  | 436 | 42.4 |  | 1.08 | 0.88 | **-** | 1.33 |  |  |
|  | AA | 86 | 11.2 |  | 130 | 12.6 |  | 0.97 | 0.71 | **-** | 1.33 |  |  |
|  |  |  |  |  |  |  |  |  |  |  |  |  | 0.85 |
| **rs3787557** | **(IVS4-763T>C)** |  |  |  |  |  |  |  |  |  |  |  |  |
| (CYP24A1-66) | TT | 558 | 71.8 |  | 740 | 71.5 |  | 1.00 |  |  |  |  |  |
|  | CT/CC | 219 | 28.2 |  | 295 | 28.5 |  | 0.97 | 0.78 | **-** | 1.19 |  |  |
|  |  |  |  |  |  |  |  |  |  |  |  |  |  |
|  |  |  |  |  |  |  |  |  |  |  |  |  | 0.77 |
| **rs2244719** | **(IVS4-486T>C)** |  |  |  |  |  |  |  |  |  |  |  |  |
| (CYP24A1-51) | TT | 192 | 25.2 |  | 266 | 26.0 |  | 1.00 |  |  |  |  |  |
|  | CT | 394 | 51.6 |  | 504 | 49.2 |  | 1.03 | 0.81 | **-** | 1.30 |  |  |
|  | CC | 177 | 23.2 |  | 254 | 24.8 |  | 0.93 | 0.71 | **-** | 1.23 |  |  |
|  |  |  |  |  |  |  |  |  |  |  |  |  | 0.62 |
| **rs3787554** | **(IVS4-308G>A)** |  |  |  |  |  |  |  |  |  |  |  |  |
| (CYP24A1-64) | GG | 623 | 80.8 |  | 850 | 82.4 |  | 1.00 |  |  |  |  |  |
|  | AG/AA | 148 | 19.2 |  | 182 | 17.6 |  | 1.07 | 0.83 | **-** | 1.36 |  |  |
|  |  |  |  |  |  |  |  |  |  |  |  |  |  |
|  |  |  |  |  |  |  |  |  |  |  |  |  | 0.48 |
| **rs3886163** | **(IVS6-792G>A)** |  |  |  |  |  |  |  |  |  |  |  |  |
| (CYP24A1-78) | GG | 626 | 80.6 |  | 828 | 80.0 |  | 1.00 |  |  |  |  |  |
|  | AG/AA | 151 | 19.4 |  | 207 | 20.0 |  | 0.96 | 0.75 | **-** | 1.22 |  |  |
|  |  |  |  |  |  |  |  |  |  |  |  |  |  |
|  |  |  |  |  |  |  |  |  |  |  |  |  | 0.81 |
| **rs912505** | **(IVS7-1179A>G)** |  |  |  |  |  |  |  |  |  |  |  |  |
| (CYP24A1-76) | AA | 474 | 61.7 |  | 643 | 62.4 |  | 1.00 |  |  |  |  |  |
|  | AG/GG | 294 | 38.3 |  | 387 | 37.6 |  | 1.01 | 0.83 | **-** | 1.23 |  |  |
|  |  |  |  |  |  |  |  |  |  |  |  |  |  |
|  |  |  |  |  |  |  |  |  |  |  |  |  | 0.62 |
| **rs1570669** | **(IVS9+198A>G)** |  |  |  |  |  |  |  |  |  |  |  |  |
| (CYP24A1-20) | AA | 307 | 39.5 |  | 400 | 38.6 |  | 1.00 |  |  |  |  |  |
|  | AG | 361 | 46.5 |  | 494 | 47.7 |  | 0.92 | 0.75 | **-** | 1.13 |  |  |
|  | GG | 109 | 14.0 |  | 141 | 13.6 |  | 1.00 | 0.74 | **-** | 1.35 |  |  |
|  |  |  |  |  |  |  |  |  |  |  |  |  | 0.76 |
| **rs927650** | **(IVS11+967C>T)** |  |  |  |  |  |  |  |  |  |  |  |  |
| (CYP24A1-77) | CC | 236 | 30.4 |  | 295 | 28.6 |  | 1.00 |  |  |  |  |  |
|  | CT | 376 | 48.5 |  | 522 | 50.5 |  | 0.88 | 0.70 | **-** | 1.10 |  |  |
|  | TT | 164 | 21.1 |  | 216 | 20.9 |  | 0.96 | 0.73 | **-** | 1.25 |  |  |
|  |  |  |  |  |  |  |  |  |  |  |  |  | 0.64 |
| **rs6097807** | **(*4262A>G)** |  |  |  |  |  |  |  |  |  |  |  |  |
| (CYP24A1-70) | AA | 436 | 56.1 |  | 593 | 57.3 |  | 1.00 |  |  |  |  |  |
|  | AG | 300 | 38.6 |  | 371 | 35.8 |  | 1.06 | 0.87 | **-** | 1.30 |  |  |
|  | GG | 41 | 5.3 |  | 71 | 6.9 |  | 0.76 | 0.51 | **-** | 1.15 |  |  |
|  |  |  |  |  |  |  |  |  |  |  |  |  | 0.64 |
| **rs6068810** | **(*4366G>T)** |  |  |  |  |  |  |  |  |  |  |  |  |
| (CYP24A1-69) | GG | 687 | 88.4 |  | 927 | 89.6 |  | 1.00 |  |  |  |  |  |
|  | GT/TT | 90 | 11.6 |  | 108 | 10.4 |  | 1.15 | 0.85 | **-** | 1.57 |  |  |
|  |  |  |  |  |  |  |  |  |  |  |  |  |  |
|  |  |  |  |  |  |  |  |  |  |  |  |  | 0.42 |
| **rs6097801** | **(Ex12+2555G>A)** |  |  |  |  |  |  |  |  |  |  |  |  |
| (CYP24A1-80) | GG | 552 | 71.0 |  | 728 | 70.3 |  | 1.00 |  |  |  |  |  |
|  | AG/AA | 225 | 29.0 |  | 307 | 29.7 |  | 0.93 | 0.75 | **-** | 1.15 |  |  |
|  |  |  |  |  |  |  |  |  |  |  |  |  |  |
|  |  |  |  |  |  |  |  |  |  |  |  |  | 0.53 |
| **rs8124792** | **(*6910G>A)** |  |  |  |  |  |  |  |  |  |  |  |  |
| (CYP24A1-75) | GG | 696 | 89.6 |  | 922 | 89.2 |  | 1.00 |  |  |  |  |  |
|  | AG/AA | 81 | 10.4 |  | 112 | 10.8 |  | 0.94 | 0.69 | **-** | 1.28 |  |  |
|  |  |  |  |  |  |  |  |  |  |  |  |  |  |
|  |  |  |  |  |  |  |  |  |  |  |  |  | 0.68 |
| **rs2762929** | **(*7522T>C)** |  |  |  |  |  |  |  |  |  |  |  |  |
| (CYP24A1-60) | TT | 302 | 39.0 |  | 419 | 40.5 |  | 1.00 |  |  |  |  |  |
|  | CT | 361 | 46.6 |  | 468 | 45.3 |  | 1.03 | 0.84 | **-** | 1.27 |  |  |
|  | CC | 112 | 14.5 |  | 147 | 14.2 |  | 1.05 | 0.78 | **-** | 1.41 |  |  |
|  |  |  |  |  |  |  |  |  |  |  |  |  | 0.70 |
| **rs6022985** | **(*8104C>G)** |  |  |  |  |  |  |  |  |  |  |  |  |
| (CYP24A1-68) | CC | 351 | 45.5 |  | 491 | 48.0 |  | 1.00 |  |  |  |  |  |
|  | CG | 362 | 46.9 |  | 437 | 42.8 |  | 1.13 | 0.93 | **-** | 1.39 |  |  |
|  | GG | 59 | 7.6 |  | 94 | 9.2 |  | 0.85 | 0.59 | **-** | 1.22 |  |  |
|  |  |  |  |  |  |  |  |  |  |  |  |  | 0.93 |
| **rs2031343** | **(*9854G>A)** |  |  |  |  |  |  |  |  |  |  |  |  |
| (CYP24A1-87) | GG | 624 | 80.8 |  | 863 | 83.7 |  | 1.00 |  |  |  |  |  |
|  | AG/AA | 148 | 19.2 |  | 168 | 16.3 |  | 1.24 | 0.97 | **-** | 1.60 |  |  |
|  |  |  |  |  |  |  |  |  |  |  |  |  |  |
|  |  |  |  |  |  |  |  |  |  |  |  |  | 0.16 |
| **rs2762927** | **(*10751C>A)** |  |  |  |  |  |  |  |  |  |  |  |  |
| (CYP24A1-59) | CC | 340 | 43.9 |  | 446 | 43.2 |  | 1.00 |  |  |  |  |  |
|  | AC | 340 | 43.9 |  | 462 | 44.7 |  | 0.94 | 0.77 | **-** | 1.15 |  |  |
|  | AA | 95 | 12.3 |  | 125 | 12.1 |  | 0.99 | 0.73 | **-** | 1.36 |  |  |
|  |  |  |  |  |  |  |  |  |  |  |  |  | 0.77 |
| **rs2585413** | **(*11237G>A)** |  |  |  |  |  |  |  |  |  |  |  |  |
| (CYP24A1-53) | GG | 395 | 51.2 |  | 519 | 50.4 |  | 1.00 |  |  |  |  |  |
|  | AG | 307 | 39.8 |  | 429 | 41.7 |  | 0.92 | 0.76 | **-** | 1.13 |  |  |
|  | AA | 70 | 9.1 |  | 81 | 7.9 |  | 1.14 | 0.80 | **-** | 1.62 |  |  |
|  |  |  |  |  |  |  |  |  |  |  |  |  | 0.96 |
| **rs9305467** | **(IVS19-5122C>T)** |  |  |  |  |  |  |  |  |  |  |  |  |
| (SFRS15-07) | CC | 685 | 88.2 |  | 893 | 86.4 |  | 1.00 |  |  |  |  |  |
|  | CT/TT | 92 | 11.8 |  | 141 | 13.6 |  | 0.89 | 0.67 | **-** | 1.19 |  |  |
|  |  |  |  |  |  |  |  |  |  |  |  |  |  |
|  |  |  |  |  |  |  |  |  |  |  |  |  | 0.44 |
| **rs2833477** | **(IVS19-4197T>C)** |  |  |  |  |  |  |  |  |  |  |  |  |
| (SFRS15-04) | TT | 601 | 77.3 |  | 804 | 77.7 |  | 1.00 |  |  |  |  |  |
|  | CT/CC | 176 | 22.7 |  | 231 | 22.3 |  | 1.02 | 0.81 | **-** | 1.28 |  |  |
|  |  |  |  |  |  |  |  |  |  |  |  |  |  |
|  |  |  |  |  |  |  |  |  |  |  |  |  | 0.81 |
| **rs2833476** | **(IVS19-2651T>C)** |  |  |  |  |  |  |  |  |  |  |  |  |
| (SFRS15-03) | TT | 672 | 86.5 |  | 905 | 87.4 |  | 1.00 |  |  |  |  |  |
|  | CT/CC | 105 | 13.5 |  | 130 | 12.6 |  | 1.11 | 0.84 | **-** | 1.48 |  |  |
|  |  |  |  |  |  |  |  |  |  |  |  |  |  |
|  |  |  |  |  |  |  |  |  |  |  |  |  | 0.61 |
| **rs202449** | **(IVS19-1616A>T)** | |  |  |  |  |  |  |  |  |  |  |  |
| (SFRS15-06) | AA | 543 | 70.6 |  | 690 | 67.1 |  | 1.00 |  |  |  |  |  |
|  | AT/TT | 226 | 29.4 |  | 338 | 32.9 |  | 0.87 | 0.71 | **-** | 1.07 |  |  |
|  |  |  |  |  |  |  |  |  |  |  |  |  |  |
|  |  |  |  |  |  |  |  |  |  |  |  |  | 0.34 |
|  |  |  |  |  |  |  |  |  |  |  |  |  |  |
| **rs16847050** | **(-17595C>T)** |  |  |  |  |  |  |  |  |  |  |  |  |
| (GC-15) | CC | 577 | 74.5 |  | 806 | 77.9 |  | 1.00 |  |  |  |  |  |
|  | CT/TT | 198 | 25.5 |  | 229 | 22.1 |  | 1.24 | 0.99 | - | 1.56 |  |  |
|  |  |  |  |  |  |  |  |  |  |  |  |  |  |
|  |  |  |  |  |  |  |  |  |  |  |  |  | **0.04** |
| **rs3733359** | **(Ex1-97G>A)** |  |  |  |  |  |  |  |  |  |  |  |  |
| (GC-12) | GG | 716 | 92.1 |  | 947 | 91.5 |  | 1.00 |  |  |  |  |  |
|  | AG/AA | 61 | 7.9 |  | 88 | 8.5 |  | 0.92 | 0.65 | **-** | 1.31 |  |  |
|  |  |  |  |  |  |  |  |  |  |  |  |  |  |
|  |  |  |  |  |  |  |  |  |  |  |  |  | 0.69 |
| **rs222029** | **(IVS1+4716A>G)** |  |  |  |  |  |  |  |  |  |  |  |  |
| (GC-10) | AA | 556 | 72.6 |  | 764 | 74.5 |  | 1.00 |  |  |  |  |  |
|  | AG/GG | 210 | 27.4 |  | 262 | 25.5 |  | 1.12 | 0.90 | **-** | 1.39 |  |  |
|  |  |  |  |  |  |  |  |  |  |  |  |  |  |
|  |  |  |  |  |  |  |  |  |  |  |  |  | 0.29 |
| **rs1352843** | **(IVS1+6724T>C)** |  |  |  |  |  |  |  |  |  |  |  |  |
| (GC-05) | TT | 622 | 80.1 |  | 858 | 82.9 |  | 1.00 |  |  |  |  |  |
|  | CT/CC | 155 | 19.9 |  | 177 | 17.1 |  | 1.21 | 0.95 | **-** | 1.55 |  |  |
|  |  |  |  |  |  |  |  |  |  |  |  |  |  |
|  |  |  |  |  |  |  |  |  |  |  |  |  | 0.21 |
| **rs16847015** | **(IVS1-3707C>A)** |  |  |  |  |  |  |  |  |  |  |  |  |
| (CG-07) | CC | 740 | 95.4 |  | 969 | 93.7 |  | 1.00 |  |  |  |  |  |
|  | AC/AA | 36 | 4.6 |  | 65 | 6.3 |  | 0.73 | 0.47 | **-** | 1.12 |  |  |
|  |  |  |  |  |  |  |  |  |  |  |  |  |  |
|  |  |  |  |  |  |  |  |  |  |  |  |  | 0.16 |
| **rs222035** | **(IVS8+755G>T)** |  |  |  |  |  |  |  |  |  |  |  |  |
| (GC-11) | GG | 248 | 32.2 |  | 328 | 31.8 |  | 1.00 |  |  |  |  |  |
|  | GT | 391 | 50.7 |  | 520 | 50.5 |  | 0.93 | 0.75 | **-** | 1.16 |  |  |
|  | TT | 132 | 17.1 |  | 182 | 17.7 |  | 0.89 | 0.67 | **-** | 1.18 |  |  |
|  |  |  |  |  |  |  |  |  |  |  |  |  | 0.38 |
| **rs1491709** | **(IVS11-1644G>A)** | |  |  |  |  |  |  |  |  |  |  |  |
| (GC-06) | GG | 710 | 91.5 |  | 931 | 90.1 |  | 1.00 |  |  |  |  |  |
|  | AG/AA | 66 | 8.5 |  | 102 | 9.9 |  | 0.82 | 0.59 | **-** | 1.15 |  |  |
|  |  |  |  |  |  |  |  |  |  |  |  |  |  |
|  |  |  |  |  |  |  |  |  |  |  |  |  | 0.30 |
| **rs705117** | **(IVS12-528T>C)** |  |  |  |  |  |  |  |  |  |  |  |  |
| (GC-13) | TT | 591 | 76.5 |  | 811 | 78.7 |  | 1.00 |  |  |  |  |  |
|  | CT/CC | 182 | 23.5 |  | 219 | 21.3 |  | 1.11 | 0.88 | **-** | 1.39 |  |  |
|  |  |  |  |  |  |  |  |  |  |  |  |  |  |
|  |  |  |  |  |  |  |  |  |  |  |  |  | 0.26 |
| **rs17467825** | **(Ex13+1894A>G)** |  |  |  |  |  |  |  |  |  |  |  |  |
| (GC-09) | AA | 376 | 49.3 |  | 485 | 47.4 |  | 1.00 |  |  |  |  |  |
|  | AG | 323 | 42.3 |  | 446 | 43.6 |  | 0.90 | 0.73 | **-** | 1.10 |  |  |
|  | GG | 64 | 8.4 |  | 93 | 9.1 |  | 0.85 | 0.60 | **-** | 1.22 |  |  |
|  |  |  |  |  |  |  |  |  |  |  |  |  | 0.24 |
| **rs17383291** | **(Ex13+2001T>G)** |  |  |  |  |  |  |  |  |  |  |  |  |
| (GC-08) | TT | 657 | 85.2 |  | 852 | 82.8 |  | 1.00 |  |  |  |  |  |
|  | GT/GG | 114 | 14.8 |  | 177 | 17.2 |  | 0.80 | 0.62 | **-** | 1.04 |  |  |
|  |  |  |  |  |  |  |  |  |  |  |  |  |  |
|  |  |  |  |  |  |  |  |  |  |  |  |  | 0.06 |
| **rs1491711** | **(*9640C>G)** |  |  |  |  |  |  |  |  |  |  |  |  |
| (GC-14) | CC | 300 | 38.7 |  | 425 | 41.1 |  | 1.00 |  |  |  |  |  |
|  | CG | 372 | 48.0 |  | 474 | 45.8 |  | 1.16 | 0.94 | **-** | 1.42 |  |  |
|  | GG | 103 | 13.3 |  | 135 | 13.1 |  | 1.18 | 0.87 | **-** | 1.61 |  |  |
|  |  |  |  |  |  |  |  |  |  |  |  |  | 0.16 |
|  |  |  |  |  |  |  |  |  |  |  |  |  |  |
| **rs3132288** | **(Ex2A>G)** |  |  |  |  |  |  |  |  |  |  |  |  |
| (LOC642985-01) | AA | 262 | 33.9 |  | 332 | 32.3 |  | 1.00 |  |  |  |  |  |
|  | AG | 363 | 47.0 |  | 491 | 47.7 |  | 0.91 | 0.73 | **-** | 1.13 |  |  |
|  | GG | 147 | 19.0 |  | 206 | 20.0 |  | 0.91 | 0.69 | **-** | 1.19 |  |  |
|  |  |  |  |  |  |  |  |  |  |  |  |  | 0.43 |
| **rs9409929** | **(*12052G>A)** |  |  |  |  |  |  |  |  |  |  |  |  |
| (RXRA-49) | GG | 302 | 38.9 |  | 415 | 40.1 |  | 1.00 |  |  |  |  |  |
|  | AG | 362 | 46.6 |  | 460 | 44.4 |  | 1.04 | 0.85 | **-** | 1.28 |  |  |
|  | AA | 113 | 14.5 |  | 160 | 15.5 |  | 0.95 | 0.71 | **-** | 1.27 |  |  |
|  |  |  |  |  |  |  |  |  |  |  |  |  | 0.90 |
| **rs1007971** | **(*7453G>C)** |  |  |  |  |  |  |  |  |  |  |  |  |
| (RXRA-12) | GG | 484 | 62.4 |  | 697 | 68.3 |  | 1.00 |  |  |  |  |  |
|  | CG/CC | 292 | 37.6 |  | 323 | 31.7 |  | 1.27 | 1.04 | - | 1.55 |  |  |
|  |  |  |  |  |  |  |  |  |  |  |  |  |  |
|  |  |  |  |  |  |  |  |  |  |  |  |  | **0.05** |
| **rs3118523** | **(*7060A>G)** |  |  |  |  |  |  |  |  |  |  |  |  |
| (RXRA-28) | AA | 482 | 62.2 |  | 714 | 69.9 |  | 1.00 |  |  |  |  |  |
|  | AG/GG | 293 | 37.8 |  | 308 | 30.1 |  | 1.40 | 1.14 | - | 1.71 |  |  |
|  |  |  |  |  |  |  |  |  |  |  |  |  |  |
|  |  |  |  |  |  |  |  |  |  |  |  |  | **0.01** |
| **rs748964** | **(*5628G>C)** |  |  |  |  |  |  |  |  |  |  |  |  |
| (RXRA-51) | GG | 563 | 72.5 |  | 811 | 78.8 |  | 1.00 |  |  |  |  |  |
|  | CG/CC | 214 | 27.5 |  | 218 | 21.2 |  | 1.42 | 1.13 | - | 1.77 |  |  |
|  |  |  |  |  |  |  |  |  |  |  |  |  |  |
|  |  |  |  |  |  |  |  |  |  |  |  |  | **0.01** |
| **rs877954** | **(IVS9+355G>A)** |  |  |  |  |  |  |  |  |  |  |  |  |
| (RXRA-48) | GG | 349 | 44.9 |  | 448 | 43.5 |  | 1.00 |  |  |  |  |  |
|  | AG | 327 | 42.1 |  | 466 | 45.3 |  | 0.92 | 0.75 | **-** | 1.13 |  |  |
|  | AA | 101 | 13.0 |  | 115 | 11.2 |  | 1.13 | 0.83 | **-** | 1.54 |  |  |
|  |  |  |  |  |  |  |  |  |  |  |  |  | 0.80 |
| **rs3132294** | **(IVS8+278C>T)** |  |  |  |  |  |  |  |  |  |  |  |  |
| (RXRA-33) | CC | 442 | 56.9 |  | 583 | 56.6 |  | 1.00 |  |  |  |  |  |
|  | CT | 275 | 35.4 |  | 394 | 38.3 |  | 0.96 | 0.78 | **-** | 1.17 |  |  |
|  | TT | 60 | 7.7 |  | 53 | 5.1 |  | 1.49 | 1.00 | **-** | 2.23 |  |  |
|  |  |  |  |  |  |  |  |  |  |  |  |  | 0.31 |
| **rs6537944** | **(IVS5-694T>C)** |  |  |  |  |  |  |  |  |  |  |  |  |
| (RXRA-53) | TT | 688 | 89.1 |  | 888 | 86.7 |  | 1.00 |  |  |  |  |  |
|  | CT/CC | 84 | 10.9 |  | 136 | 13.3 |  | 0.78 | 0.58 | **-** | 1.05 |  |  |
|  |  |  |  |  |  |  |  |  |  |  |  |  |  |
|  |  |  |  |  |  |  |  |  |  |  |  |  | 0.15 |
| **rs4240705** | **(IVS5-2122A>G)** |  |  |  |  |  |  |  |  |  |  |  |  |
| (RXRA-38) | AA | 328 | 42.3 |  | 425 | 41.3 |  | 1.00 |  |  |  |  |  |
|  | AG | 336 | 43.3 |  | 479 | 46.5 |  | 0.92 | 0.75 | **-** | 1.13 |  |  |
|  | GG | 112 | 14.4 |  | 126 | 12.2 |  | 1.15 | 0.86 | **-** | 1.56 |  |  |
|  |  |  |  |  |  |  |  |  |  |  |  |  | 0.69 |
| **rs3118536** | **(IVS4-542C>A)** |  |  |  |  |  |  |  |  |  |  |  |  |
| (RXRA-31) | CC | 518 | 66.7 |  | 725 | 70.3 |  | 1.00 |  |  |  |  |  |
|  | AC/AA | 259 | 33.3 |  | 306 | 29.7 |  | 1.22 | 1.00 | - | 1.50 |  |  |
|  |  |  |  |  |  |  |  |  |  |  |  |  |  |
|  |  |  |  |  |  |  |  |  |  |  |  |  | **0.02** |
| **rs3132296** | **(IVS4+1666T>C)** | |  |  |  |  |  |  |  |  |  |  |  |
| (RXRA-34) | TT | 359 | 46.2 |  | 488 | 47.5 |  | 1.00 |  |  |  |  |  |
|  | CT | 322 | 41.4 |  | 438 | 42.6 |  | 1.03 | 0.84 | **-** | 1.26 |  |  |
|  | CC | 96 | 12.4 |  | 102 | 9.9 |  | 1.31 | 0.96 | **-** | 1.81 |  |  |
|  |  |  |  |  |  |  |  |  |  |  |  |  | 0.16 |
| **rs10776909** | **(IVS1-4732C>T)** |  |  |  |  |  |  |  |  |  |  |  |  |
| (RXRA-15) | CC | 475 | 61.2 |  | 663 | 64.1 |  | 1.00 |  |  |  |  |  |
|  | CT/TT | 301 | 38.8 |  | 372 | 35.9 |  | 1.17 | 0.96 | - | 1.42 |  |  |
|  |  |  |  |  |  |  |  |  |  |  |  |  |  |
|  |  |  |  |  |  |  |  |  |  |  |  |  | **0.05** |
| **rs11103473** | **(IVS1-5849A>T)** | |  |  |  |  |  |  |  |  |  |  |  |
| (RXRA-19) | AA | 316 | 40.7 |  | 433 | 41.9 |  | 1.00 |  |  |  |  |  |
|  | AT | 345 | 44.4 |  | 470 | 45.5 |  | 1.04 | 0.85 | **-** | 1.28 |  |  |
|  | TT | 116 | 14.9 |  | 130 | 12.6 |  | 1.27 | 0.94 | **-** | 1.71 |  |  |
|  |  |  |  |  |  |  |  |  |  |  |  |  | 0.16 |
| **rs7039190** | **(IVS1-26774A>C)** | |  |  |  |  |  |  |  |  |  |  |  |
| (RXRA-54) | AA | 692 | 89.1 |  | 905 | 87.4 |  | 1.00 |  |  |  |  |  |
|  | AC/CC | 85 | 10.9 |  | 130 | 12.6 |  | 0.87 | 0.65 | **-** | 1.18 |  |  |
|  |  |  |  |  |  |  |  |  |  |  |  |  |  |
|  |  |  |  |  |  |  |  |  |  |  |  |  | 0.42 |
| **rs11185662** | **(IVS1-31359T>C)** |  |  |  |  |  |  |  |  |  |  |  |  |
| (RXRA-52) | TT | 453 | 58.4 |  | 601 | 58.4 |  | 1.00 |  |  |  |  |  |
|  | CT | 275 | 35.4 |  | 375 | 36.4 |  | 0.97 | 0.79 | **-** | 1.18 |  |  |
|  | CC | 48 | 6.2 |  | 53 | 5.2 |  | 1.30 | 0.85 | **-** | 1.99 |  |  |
|  |  |  |  |  |  |  |  |  |  |  |  |  | 0.58 |
| **rs7871655** | **(IVS1+34281G>C)** | |  |  |  |  |  |  |  |  |  |  |  |
| (RXRA-55) | GG | 432 | 55.6 |  | 590 | 57.5 |  | 1.00 |  |  |  |  |  |
|  | CG/CC | 345 | 44.4 |  | 436 | 42.5 |  | 1.06 | 0.87 | **-** | 1.29 |  |  |
|  |  |  |  |  |  |  |  |  |  |  |  |  |  |
|  |  |  |  |  |  |  |  |  |  |  |  |  | 0.27 |
| **rs881658** | **(IVS1+11350C>T)** | |  |  |  |  |  |  |  |  |  |  |  |
| (RXRA-56) | CC | 352 | 45.4 |  | 468 | 45.3 |  | 1.00 |  |  |  |  |  |
|  | CT | 346 | 44.6 |  | 466 | 45.1 |  | 0.97 | 0.79 | **-** | 1.18 |  |  |
|  | TT | 78 | 10.1 |  | 99 | 9.6 |  | 1.05 | 0.75 | **-** | 1.47 |  |  |
|  |  |  |  |  |  |  |  |  |  |  |  |  | 0.97 |
| **rs4917348** | **(-4332A>G)** |  |  |  |  |  |  |  |  |  |  |  |  |
| (RXRA-58) | AA | 516 | 66.4 |  | 698 | 67.6 |  | 1.00 |  |  |  |  |  |
|  | AG/GG | 261 | 33.6 |  | 335 | 32.4 |  | 1.06 | 0.86 | **-** | 1.30 |  |  |
|  |  |  |  |  |  |  |  |  |  |  |  |  |  |
|  |  |  |  |  |  |  |  |  |  |  |  |  | 0.52 |
|  |  |  |  |  |  |  |  |  |  |  |  |  |  |
| **rs9277936** | **(*3071T>A)** |  |  |  |  |  |  |  |  |  |  |  |  |
| (RING1-07) | TT | 377 | 48.5 |  | 543 | 52.5 |  | 1.00 |  |  |  |  |  |
|  | AT | 335 | 43.1 |  | 412 | 39.8 |  | 1.16 | 0.95 | **-** | 1.41 |  |  |
|  | AA | 65 | 8.4 |  | 80 | 7.7 |  | 1.13 | 0.79 | **-** | 1.62 |  |  |
|  |  |  |  |  |  |  |  |  |  |  |  |  | 0.20 |
| **rs2854028** | **(Ex6-91C>T)** |  |  |  |  |  |  |  |  |  |  |  |  |
| (RING1-10) | CC | 403 | 51.9 |  | 588 | 56.9 |  | 1.00 |  |  |  |  |  |
|  | CT | 324 | 41.7 |  | 385 | 37.2 |  | 1.22 | 1.00 | **-** | 1.49 |  |  |
|  | TT | 50 | 6.4 |  | 61 | 5.9 |  | 1.15 | 0.77 | **-** | 1.72 |  |  |
|  |  |  |  |  |  |  |  |  |  |  |  |  | 0.09 |
| **rs421446** | **(*351A>G)** |  |  |  |  |  |  |  |  |  |  |  |  |
| (HSD17B8-01) | AA | 428 | 55.5 |  | 584 | 56.8 |  | 1.00 |  |  |  |  |  |
|  | AG | 296 | 38.4 |  | 382 | 37.1 |  | 1.05 | 0.86 | **-** | 1.29 |  |  |
|  | GG | 47 | 6.1 |  | 63 | 6.1 |  | 0.95 | 0.63 | **-** | 1.43 |  |  |
|  |  |  |  |  |  |  |  |  |  |  |  |  | 0.88 |
| **rs1547387** | **(Ex3-8C>G)** |  |  |  |  |  |  |  |  |  |  |  |  |
| (SLC39A7-01) | CC | 583 | 75.0 |  | 779 | 75.6 |  | 1.00 |  |  |  |  |  |
|  | CG/GG | 194 | 25.0 |  | 252 | 24.4 |  | 1.04 | 0.83 | **-** | 1.29 |  |  |
|  |  |  |  |  |  |  |  |  |  |  |  |  |  |
|  |  |  |  |  |  |  |  |  |  |  |  |  | 0.59 |
| **rs6531** | **(Ex7+29A>G)** |  |  |  |  |  |  |  |  |  |  |  |  |
| (RXRB-13) | AA | 446 | 57.4 |  | 562 | 54.5 |  | 1.00 |  |  |  |  |  |
|  | AG | 287 | 36.9 |  | 400 | 38.8 |  | 0.90 | 0.74 | **-** | 1.10 |  |  |
|  | GG | 44 | 5.7 |  | 70 | 6.8 |  | 0.82 | 0.55 | **-** | 1.23 |  |  |
|  |  |  |  |  |  |  |  |  |  |  |  |  | 0.20 |
| **rs2269346** | **(IVS1+1038C>T)** |  |  |  |  |  |  |  |  |  |  |  |  |
| (COL11A2-02) | CC | 715 | 92.0 |  | 941 | 91.2 |  | 1.00 |  |  |  |  |  |
|  | CT/TT | 62 | 8.0 |  | 91 | 8.8 |  | 0.91 | 0.64 | **-** | 1.29 |  |  |
|  |  |  |  |  |  |  |  |  |  |  |  |  |  |
|  |  |  |  |  |  |  |  |  |  |  |  |  | 0.60 |
| **rs2855459** | **(IVS4-61G>A)** |  |  |  |  |  |  |  |  |  |  |  |  |
| (COL11A2-07) | GG | 612 | 78.9 |  | 830 | 80.7 |  | 1.00 |  |  |  |  |  |
|  | AG/AA | 164 | 21.1 |  | 199 | 19.3 |  | 1.05 | 0.83 | **-** | 1.33 |  |  |
|  |  |  |  |  |  |  |  |  |  |  |  |  |  |
|  |  |  |  |  |  |  |  |  |  |  |  |  | 0.58 |
| **rs9277934** | **(Ex6+28C>T)** |  |  |  |  |  |  |  |  |  |  |  |  |
| (COL11A2-05) | CC | 324 | 41.8 |  | 466 | 45.0 |  | 1.00 |  |  |  |  |  |
|  | CT | 368 | 47.5 |  | 448 | 43.3 |  | 1.17 | 0.96 | **-** | 1.44 |  |  |
|  | TT | 83 | 10.7 |  | 121 | 11.7 |  | 0.97 | 0.70 | **-** | 1.34 |  |  |
|  |  |  |  |  |  |  |  |  |  |  |  |  | 0.56 |
|  |  |  |  |  |  |  |  |  |  |  |  |  |  |
| **rs925847** | **(IVS21+144C>T)** |  |  |  |  |  |  |  |  |  |  |  |  |
| (STAT4-36) | CC | 417 | 53.7 |  | 620 | 60.0 |  | 1.00 |  |  |  |  |  |
|  | CT | 301 | 38.8 |  | 338 | 32.7 |  | 1.32 | 1.07 | **-** | 1.61 |  |  |
|  | TT | 58 | 7.5 |  | 75 | 7.3 |  | 1.10 | 0.75 | **-** | 1.60 |  |  |
|  |  |  |  |  |  |  |  |  |  |  |  |  | 0.06 |
| **rs3024896** | **(IVS21-474C>T)** |  |  |  |  |  |  |  |  |  |  |  |  |
| (STAT4-24) | CC | 526 | 68.6 |  | 747 | 73.5 |  | 1.00 |  |  |  |  |  |
|  | CT/TT | 241 | 31.4 |  | 270 | 26.5 |  | 1.23 | 1.00 | **-** | 1.52 |  |  |
|  |  |  |  |  |  |  |  |  |  |  |  |  |  |
|  |  |  |  |  |  |  |  |  |  |  |  |  | 0.19 |
| **rs3024936** | **(IVS23+322G>C)** |  |  |  |  |  |  |  |  |  |  |  |  |
| (STAT4-28) | GG | 735 | 94.6 |  | 978 | 94.5 |  | 1.00 |  |  |  |  |  |
|  | CG | 42 | 5.4 |  | 57 | 5.5 |  | 1.06 | 0.70 | **-** | 1.61 |  |  |
|  |  |  |  |  |  |  |  |  |  |  |  |  |  |
|  |  |  |  |  |  |  |  |  |  |  |  |  | 0.80 |
| **rs6740131** | **(*4983A>G)** |  |  |  |  |  |  |  |  |  |  |  |  |
| (STAT4-45) | AA | 470 | 60.5 |  | 634 | 61.3 |  | 1.00 |  |  |  |  |  |
|  | AG/GG | 307 | 39.5 |  | 400 | 38.7 |  | 1.07 | 0.88 | **-** | 1.30 |  |  |
|  |  |  |  |  |  |  |  |  |  |  |  |  |  |
|  |  |  |  |  |  |  |  |  |  |  |  |  | 0.61 |
| **rs7558921** | **(*6341G>C)** |  |  |  |  |  |  |  |  |  |  |  |  |
| (STAT4-46) | GG | 593 | 76.3 |  | 811 | 78.4 |  | 1.00 |  |  |  |  |  |
|  | CG/CC | 184 | 23.7 |  | 224 | 21.6 |  | 1.16 | 0.93 | **-** | 1.46 |  |  |
|  |  |  |  |  |  |  |  |  |  |  |  |  |  |
|  |  |  |  |  |  |  |  |  |  |  |  |  | 0.20 |
| **rs6751855** | **( -10041A>G)** |  |  |  |  |  |  |  |  |  |  |  |  |
| (STAT1-23) | AA | 277 | 36.7 |  | 424 | 41.9 |  | 1.00 |  |  |  |  |  |
|  | AG | 316 | 41.9 |  | 411 | 40.6 |  | 1.15 | 0.92 | - | 1.42 |  |  |
|  | GG | 161 | 21.4 |  | 178 | 17.6 |  | 1.36 | 1.04 | - | 1.78 |  |  |
|  |  |  |  |  |  |  |  |  |  |  |  |  | **0.02** |
| **rs1467199** | **( -5772C>G)** |  |  |  |  |  |  |  |  |  |  |  |  |
| (STAT1-22) | CC | 496 | 64.0 |  | 694 | 67.3 |  | 1.00 |  |  |  |  |  |
|  | CG/GG | 279 | 36.0 |  | 337 | 32.7 |  | 1.15 | 0.94 | **-** | 1.41 |  |  |
|  |  |  |  |  |  |  |  |  |  |  |  |  |  |
|  |  |  |  |  |  |  |  |  |  |  |  |  | 0.12 |
| **rs13029532** | **(IVS2-1171A>C)** |  |  |  |  |  |  |  |  |  |  |  |  |
| (STAT1-14) | AA | 631 | 81.2 |  | 861 | 83.2 |  | 1.00 |  |  |  |  |  |
|  | AC/CC | 146 | 18.8 |  | 174 | 16.8 |  | 1.16 | 0.90 | **-** | 1.48 |  |  |
|  |  |  |  |  |  |  |  |  |  |  |  |  |  |
|  |  |  |  |  |  |  |  |  |  |  |  |  | 0.20 |
| **rs13029247** | **(IVS5-769T>C)** |  |  |  |  |  |  |  |  |  |  |  |  |
| (STAT1-24) | TT | 418 | 54.6 |  | 586 | 57.1 |  | 1.00 |  |  |  |  |  |
|  | CT | 287 | 37.5 |  | 367 | 35.7 |  | 1.12 | 0.91 | **-** | 1.37 |  |  |
|  | CC | 61 | 8.0 |  | 74 | 7.2 |  | 1.11 | 0.76 | **-** | 1.61 |  |  |
|  |  |  |  |  |  |  |  |  |  |  |  |  | 0.32 |
| **rs12693591** | **(IVS9-557C>A)** |  |  |  |  |  |  |  |  |  |  |  |  |
| (STAT1-11) | CC | 592 | 76.3 |  | 791 | 76.4 |  | 1.00 |  |  |  |  |  |
|  | AC/AA | 184 | 23.7 |  | 244 | 23.6 |  | 0.99 | 0.79 | **-** | 1.24 |  |  |
|  |  |  |  |  |  |  |  |  |  |  |  |  |  |
|  |  |  |  |  |  |  |  |  |  |  |  |  | 0.92 |
| **rs7562024** | **(IVS11+433C>T)** |  |  |  |  |  |  |  |  |  |  |  |  |
| (STAT1-20) | CC | 290 | 37.4 |  | 431 | 41.8 |  | 1.00 |  |  |  |  |  |
|  | CT | 360 | 46.5 |  | 453 | 43.9 |  | 1.18 | 0.96 | **-** | 1.45 |  |  |
|  | TT | 125 | 16.1 |  | 148 | 14.3 |  | 1.21 | 0.91 | **-** | 1.62 |  |  |
|  |  |  |  |  |  |  |  |  |  |  |  |  | 0.11 |
| **rs13005843** | **(IVS11-326C>T)** |  |  |  |  |  |  |  |  |  |  |  |  |
| (STAT1-12) | CC | 676 | 87.1 |  | 898 | 87.0 |  | 1.00 |  |  |  |  |  |
|  | CT/TT | 100 | 12.9 |  | 134 | 13.0 |  | 1.01 | 0.76 | **-** | 1.34 |  |  |
|  |  |  |  |  |  |  |  |  |  |  |  |  |  |
|  |  |  |  |  |  |  |  |  |  |  |  |  | 0.99 |
| **rs2280232** | **(IVS14-380A>C)** |  |  |  |  |  |  |  |  |  |  |  |  |
| (STAT1-25) | AA | 428 | 55.1 |  | 612 | 59.2 |  | 1.00 |  |  |  |  |  |
|  | AC | 303 | 39.0 |  | 363 | 35.1 |  | 1.23 | 1.00 | **-** | 1.50 |  |  |
|  | CC | 46 | 5.9 |  | 59 | 5.7 |  | 1.08 | 0.71 | **-** | 1.64 |  |  |
|  |  |  |  |  |  |  |  |  |  |  |  |  | 0.12 |
| **rs1547550** | **(IVS18-330C>G)** |  |  |  |  |  |  |  |  |  |  |  |  |
| (STAT1-16) | CC | 332 | 42.7 |  | 471 | 45.6 |  | 1.00 |  |  |  |  |  |
|  | CG | 352 | 45.3 |  | 433 | 41.9 |  | 1.12 | 0.91 | **-** | 1.38 |  |  |
|  | GG | 93 | 12.0 |  | 129 | 12.5 |  | 1.00 | 0.74 | **-** | 1.37 |  |  |
|  |  |  |  |  |  |  |  |  |  |  |  |  | 0.62 |
| **rs13010343** | **(IVS21+137G>A)** |  |  |  |  |  |  |  |  |  |  |  |  |
| (STAT1-13) | GG | 596 | 76.7 |  | 812 | 78.5 |  | 1.00 |  |  |  |  |  |
|  | AG/AA | 181 | 23.3 |  | 223 | 21.5 |  | 1.12 | 0.89 | **-** | 1.41 |  |  |
|  |  |  |  |  |  |  |  |  |  |  |  |  |  |
|  |  |  |  |  |  |  |  |  |  |  |  |  | 0.46 |
| **rs2066804** | **(IVS21-8G>A)** |  |  |  |  |  |  |  |  |  |  |  |  |
| (STAT1-01) | GG | 484 | 62.5 |  | 649 | 62.9 |  | 1.00 |  |  |  |  |  |
|  | AG | 248 | 32.0 |  | 330 | 32.0 |  | 1.01 | 0.82 | **-** | 1.24 |  |  |
|  | AA | 42 | 5.4 |  | 53 | 5.1 |  | 0.99 | 0.64 | **-** | 1.53 |  |  |
|  |  |  |  |  |  |  |  |  |  |  |  |  | 0.98 |
| **rs17749316** | **(IVS22-96C>G)** |  |  |  |  |  |  |  |  |  |  |  |  |
| (STAT1-17) | CC | 656 | 84.4 |  | 848 | 82.0 |  | 1.00 |  |  |  |  |  |
|  | CG/GG | 121 | 15.6 |  | 186 | 18.0 |  | 0.87 | 0.68 | **-** | 1.13 |  |  |
|  |  |  |  |  |  |  |  |  |  |  |  |  |  |
|  |  |  |  |  |  |  |  |  |  |  |  |  | 0.19 |
| **rs16824035** | **(IVS24+1922C>T)** | |  |  |  |  |  |  |  |  |  |  |  |
| (STAT1-27) | CC | 559 | 72.0 |  | 760 | 73.5 |  | 1.00 |  |  |  |  |  |
|  | CT/TT | 217 | 28.0 |  | 274 | 26.5 |  | 1.10 | 0.89 | **-** | 1.37 |  |  |
|  |  |  |  |  |  |  |  |  |  |  |  |  |  |
|  |  |  |  |  |  |  |  |  |  |  |  |  | 0.56 |
| **rs3771300** | **(IVS24-153G>T)** |  |  |  |  |  |  |  |  |  |  |  |  |
| (STA1-18) | GG | 215 | 27.7 |  | 312 | 30.2 |  | 1.00 |  |  |  |  |  |
|  | GT | 390 | 50.3 |  | 513 | 49.6 |  | 1.16 | 0.93 | **-** | 1.45 |  |  |
|  | TT | 171 | 22.0 |  | 209 | 20.2 |  | 1.17 | 0.89 | **-** | 1.53 |  |  |
|  |  |  |  |  |  |  |  |  |  |  |  |  | 0.23 |
| **rs12468579** | **(*3164A>G)** |  |  |  |  |  |  |  |  |  |  |  |  |
| (STAT1-26) | AA | 290 | 37.3 |  | 392 | 37.9 |  | 1.00 |  |  |  |  |  |
|  | AG | 363 | 46.7 |  | 482 | 46.7 |  | 1.05 | 0.85 | **-** | 1.30 |  |  |
|  | GG | 124 | 16.0 |  | 159 | 15.4 |  | 1.01 | 0.76 | **-** | 1.35 |  |  |
|  |  |  |  |  |  |  |  |  |  |  |  |  | 0.84 |
| **rs883844** | **(IVS17-1787C>T)** |  |  |  |  |  |  |  |  |  |  |  |  |
| (GLS-02) | CC | 349 | 45.6 |  | 496 | 48.3 |  | 1.00 |  |  |  |  |  |
|  | CT | 322 | 42.0 |  | 415 | 40.4 |  | 1.11 | 0.91 |  | 1.37 |  |  |
|  | TT | 95 | 12.4 |  | 115 | 11.2 |  | 1.12 | 0.82 |  | 1.54 |  |  |
|  |  |  |  |  |  |  |  |  |  |  |  |  | 0.31 |
|  |  |  |  |  |  |  |  |  |  |  |  |  |  |
| **rs1568400** | **(IVS1+1763T>C)** |  |  |  |  |  |  |  |  |  |  |  |  |
| (THRA-02) | TT | 489 | 63.0 |  | 647 | 62.6 |  | 1.00 |  |  |  |  |  |
|  | CT/CC | 287 | 37.0 |  | 387 | 37.4 |  | 0.99 | 0.82 | **-** | 1.21 |  |  |
|  |  |  |  |  |  |  |  |  |  |  |  |  |  |
|  |  |  |  |  |  |  |  |  |  |  |  |  | 0.89 |
| **rs7502514** | **(IVS8+58G>A)** |  |  |  |  |  |  |  |  |  |  |  |  |
| (THRAP4-11) | GG | 294 | 37.8 |  | 383 | 37.2 |  | 1.00 |  |  |  |  |  |
|  | AG | 368 | 47.4 |  | 497 | 48.3 |  | 0.97 | 0.79 | **-** | 1.20 |  |  |
|  | AA | 115 | 14.8 |  | 149 | 14.5 |  | 1.03 | 0.77 | **-** | 1.38 |  |  |
|  |  |  |  |  |  |  |  |  |  |  |  |  | 0.94 |
| **rs2302775** | **(IVS17+56T>C)** |  |  |  |  |  |  |  |  |  |  |  |  |
| (THRAP4-10) | TT | 492 | 63.4 |  | 651 | 63.0 |  | 1.00 |  |  |  |  |  |
|  | CT/CC | 284 | 36.6 |  | 383 | 37.0 |  | 0.96 | 0.79 | **-** | 1.17 |  |  |
|  |  |  |  |  |  |  |  |  |  |  |  |  |  |
|  |  |  |  |  |  |  |  |  |  |  |  |  | 0.86 |
| **rs9916158** | **(IVS18+180G>T)** |  |  |  |  |  |  |  |  |  |  |  |  |
| (THRAP4-09) | GG | 265 | 34.1 |  | 364 | 35.2 |  | 1.00 |  |  |  |  |  |
|  | GT | 395 | 50.9 |  | 510 | 49.3 |  | 1.04 | 0.84 | **-** | 1.29 |  |  |
|  | TT | 116 | 14.9 |  | 160 | 15.5 |  | 0.98 | 0.73 | **-** | 1.31 |  |  |
|  |  |  |  |  |  |  |  |  |  |  |  |  | 0.99 |
| **rs9913632** | **(IVS22+776C>T)** |  |  |  |  |  |  |  |  |  |  |  |  |
| (THRAP4-07) | CC | 686 | 88.3 |  | 926 | 89.6 |  | 1.00 |  |  |  |  |  |
|  | CT/TT | 91 | 11.7 |  | 108 | 10.4 |  | 1.13 | 0.83 | **-** | 1.53 |  |  |
|  |  |  |  |  |  |  |  |  |  |  |  |  |  |
|  |  |  |  |  |  |  |  |  |  |  |  |  | 0.58 |
| **rs2827** | **(Ex4-330C>T)** |  |  |  |  |  |  |  |  |  |  |  |  |
| (CSF3-20) | CC | 570 | 73.5 |  | 752 | 72.9 |  | 1.00 |  |  |  |  |  |
|  | CT/TT | 206 | 26.5 |  | 280 | 27.1 |  | 0.95 | 0.77 | **-** | 1.18 |  |  |
|  |  |  |  |  |  |  |  |  |  |  |  |  |  |
|  |  |  |  |  |  |  |  |  |  |  |  |  | 0.65 |
|  |  |  |  |  |  |  |  |  |  |  |  |  |  |
| **rs168405** | **(IVS1+2345T>G)** |  |  |  |  |  |  |  |  |  |  |  |  |
| (C19ORF22-01) | TT | 348 | 44.8 |  | 454 | 44.0 |  | 1.00 |  |  |  |  |  |
|  | GT | 351 | 45.2 |  | 454 | 44.0 |  | 1.06 | 0.87 | **-** | 1.30 |  |  |
|  | GG | 78 | 10.0 |  | 123 | 11.9 |  | 0.85 | 0.62 | **-** | 1.18 |  |  |
|  |  |  |  |  |  |  |  |  |  |  |  |  | 0.66 |
| **rs2306718** | **(IVS5+83G>A)** |  |  |  |  |  |  |  |  |  |  |  |  |
| (C19ORF22-02) | GG | 440 | 56.6 |  | 588 | 57.2 |  | 1.00 |  |  |  |  |  |
|  | AG | 298 | 38.4 |  | 361 | 35.1 |  | 1.11 | 0.91 | **-** | 1.36 |  |  |
|  | AA | 39 | 5.0 |  | 79 | 7.7 |  | 0.68 | 0.45 | **-** | 1.03 |  |  |
|  |  |  |  |  |  |  |  |  |  |  |  |  | 0.58 |
| **rs2965286** | **(*937C>T)** |  |  |  |  |  |  |  |  |  |  |  |  |
| (C19ORF22-03) | CC | 493 | 63.5 |  | 644 | 62.4 |  | 1.00 |  |  |  |  |  |
|  | CT/TT | 283 | 36.5 |  | 388 | 37.6 |  | 0.99 | 0.81 | **-** | 1.20 |  |  |
|  |  |  |  |  |  |  |  |  |  |  |  |  |  |
|  |  |  |  |  |  |  |  |  |  |  |  |  | 0.72 |
| **rs2930902** | **(IVS4-927A>G)** |  |  |  |  |  |  |  |  |  |  |  |  |
| (THRAP5-10) | AA | 520 | 66.9 |  | 684 | 66.5 |  | 1.00 |  |  |  |  |  |
|  | AG/GG | 257 | 33.1 |  | 344 | 33.5 |  | 1.02 | 0.83 | **-** | 1.25 |  |  |
|  |  |  |  |  |  |  |  |  |  |  |  |  |  |
|  |  |  |  |  |  |  |  |  |  |  |  |  | 0.99 |
| **rs1060442** | **(Ex5-49G>A)** |  |  |  |  |  |  |  |  |  |  |  |  |
| (THRAP5-06) | GG | 248 | 31.9 |  | 347 | 33.8 |  | 1.00 |  |  |  |  |  |
|  | AG | 387 | 49.8 |  | 500 | 48.6 |  | 1.06 | 0.86 | **-** | 1.32 |  |  |
|  | AA | 142 | 18.3 |  | 181 | 17.6 |  | 1.15 | 0.87 | **-** | 1.53 |  |  |
|  |  |  |  |  |  |  |  |  |  |  |  |  | 0.32 |
| **rs2965294** | **(IVS6+121C>G)** |  |  |  |  |  |  |  |  |  |  |  |  |
| (THRAP5-11) | CC | 272 | 35.1 |  | 327 | 31.7 |  | 1.00 |  |  |  |  |  |
|  | CG | 374 | 48.2 |  | 522 | 50.7 |  | 0.86 | 0.70 | **-** | 1.07 |  |  |
|  | GG | 130 | 16.8 |  | 181 | 17.6 |  | 0.87 | 0.66 | **-** | 1.16 |  |  |
|  |  |  |  |  |  |  |  |  |  |  |  |  | 0.25 |
| **rs1617214** | **(IVS8-257G>C)** |  |  |  |  |  |  |  |  |  |  |  |  |
| (THRAP5-08) | GG | 446 | 57.5 |  | 551 | 55.5 |  | 1.00 |  |  |  |  |  |
|  | CG | 282 | 36.4 |  | 381 | 38.4 |  | 0.91 | 0.75 | **-** | 1.12 |  |  |
|  | CC | 47 | 6.1 |  | 60 | 6.0 |  | 1.00 | 0.66 | **-** | 1.51 |  |  |
|  |  |  |  |  |  |  |  |  |  |  |  |  | 0.57 |
| **rs2241623** | **(IVS9+370C>T)** |  |  |  |  |  |  |  |  |  |  |  |  |
| (THRAP5-09) | CC | 352 | 45.3 |  | 470 | 45.7 |  | 1.00 |  |  |  |  |  |
|  | CT | 353 | 45.4 |  | 444 | 43.2 |  | 1.05 | 0.86 | **-** | 1.28 |  |  |
|  | TT | 72 | 9.3 |  | 114 | 11.1 |  | 0.82 | 0.59 | **-** | 1.15 |  |  |
|  |  |  |  |  |  |  |  |  |  |  |  |  | 0.54 |
| **rs13090** | **(Ex16+137C>T)** |  |  |  |  |  |  |  |  |  |  |  |  |
| (THRAP5-07) | CC | 441 | 56.8 |  | 569 | 55.1 |  | 1.00 |  |  |  |  |  |
|  | CT | 282 | 36.3 |  | 394 | 38.2 |  | 0.93 | 0.76 | **-** | 1.14 |  |  |
|  | TT | 53 | 6.8 |  | 69 | 6.7 |  | 0.99 | 0.67 | **-** | 1.46 |  |  |
|  |  |  |  |  |  |  |  |  |  |  |  |  | 0.63 |
| **rs17684161** | **(*2694T>C)** |  |  |  |  |  |  |  |  |  |  |  |  |
| (THRAP5-05) | TT | 597 | 76.8 |  | 768 | 74.4 |  | 1.00 |  |  |  |  |  |
|  | CT/CC | 180 | 23.2 |  | 264 | 25.6 |  | 0.86 | 0.69 | **-** | 1.08 |  |  |
|  |  |  |  |  |  |  |  |  |  |  |  |  |  |
|  |  |  |  |  |  |  |  |  |  |  |  |  | 0.11 |
| **rs1683564** | **( -475C>A)** |  |  |  |  |  |  |  |  |  |  |  |  |
| (DF-03) | CC | 290 | 37.3 |  | 386 | 37.5 |  | 1.00 |  |  |  |  |  |
|  | AC | 388 | 49.9 |  | 490 | 47.6 |  | 1.06 | 0.86 | **-** | 1.30 |  |  |
|  | AA | 99 | 12.7 |  | 153 | 14.9 |  | 0.89 | 0.66 | **-** | 1.21 |  |  |
|  |  |  |  |  |  |  |  |  |  |  |  |  | 0.69 |
|  |  |  |  |  |  |  |  |  |  |  |  |  |  |
| **rs10459228** | **( -43825C>T)** |  |  |  |  |  |  |  |  |  |  |  |  |
| (VDR-110) | CC | 565 | 72.7 |  | 732 | 70.7 |  | 1.00 |  |  |  |  |  |
|  | CT/TT | 212 | 27.3 |  | 303 | 29.3 |  | 0.90 | 0.73 | **-** | 1.12 |  |  |
|  |  |  |  |  |  |  |  |  |  |  |  |  |  |
|  |  |  |  |  |  |  |  |  |  |  |  |  | 0.17 |
| **rs4516035** | **( -26929T>C)** |  |  |  |  |  |  |  |  |  |  |  |  |
| (VDR-19) | TT | 235 | 30.2 |  | 322 | 31.1 |  | 1.00 |  |  |  |  |  |
|  | CT | 403 | 51.9 |  | 527 | 51.0 |  | 1.09 | 0.87 | **-** | 1.36 |  |  |
|  | CC | 139 | 17.9 |  | 185 | 17.9 |  | 1.09 | 0.82 | **-** | 1.45 |  |  |
|  |  |  |  |  |  |  |  |  |  |  |  |  | 0.51 |
| **rs10783219** | **(IVS1-1747A>T)** |  |  |  |  |  |  |  |  |  |  |  |  |
| (VDR-105) | AA | 278 | 35.8 |  | 357 | 34.6 |  | 1.00 |  |  |  |  |  |
|  | AT | 381 | 49.0 |  | 512 | 49.6 |  | 0.94 | 0.77 | **-** | 1.17 |  |  |
|  | TT | 118 | 15.2 |  | 163 | 15.8 |  | 0.88 | 0.66 | **-** | 1.18 |  |  |
|  |  |  |  |  |  |  |  |  |  |  |  |  | 0.39 |
| **rs11168292** | **(IVS2+15C>G)** |  |  |  |  |  |  |  |  |  |  |  |  |
| (VDR-112) | CC | 331 | 42.6 |  | 467 | 45.2 |  | 1.00 |  |  |  |  |  |
|  | CG | 363 | 46.7 |  | 450 | 43.6 |  | 1.17 | 0.96 | **-** | 1.43 |  |  |
|  | GG | 83 | 10.7 |  | 116 | 11.2 |  | 1.08 | 0.78 | **-** | 1.50 |  |  |
|  |  |  |  |  |  |  |  |  |  |  |  |  | 0.28 |
| **rs10875695** | **(IVS2+583C>A)** |  |  |  |  |  |  |  |  |  |  |  |  |
| (VDR-106) | CC | 472 | 61.1 |  | 613 | 59.4 |  | 1.00 |  |  |  |  |  |
|  | AC | 261 | 33.8 |  | 355 | 34.4 |  | 0.95 | 0.77 | **-** | 1.17 |  |  |
|  | AA | 39 | 5.1 |  | 64 | 6.2 |  | 0.79 | 0.52 | **-** | 1.21 |  |  |
|  |  |  |  |  |  |  |  |  |  |  |  |  | 0.31 |
| **rs11574026** | **(IVS2+5374G>A)** |  |  |  |  |  |  |  |  |  |  |  |  |
| (VDR-25) | GG | 645 | 83.0 |  | 859 | 83.0 |  | 1.00 |  |  |  |  |  |
|  | AG/AA | 132 | 17.0 |  | 176 | 17.0 |  | 0.99 | 0.77 | **-** | 1.28 |  |  |
|  |  |  |  |  |  |  |  |  |  |  |  |  |  |
|  |  |  |  |  |  |  |  |  |  |  |  |  | 0.90 |
| **rs11574027** | **(IVS2+6247C>A)** |  |  |  |  |  |  |  |  |  |  |  |  |
| (VDR-107) | CC | 752 | 96.8 |  | 1018 | 98.4 |  | 1.00 |  |  |  |  |  |
|  | AC | 25 | 3.2 |  | 17 | 1.6 |  | 1.99 | 1.05 | - | 3.77 |  |  |
|  |  |  |  |  |  |  |  |  |  |  |  |  |  |
|  |  |  |  |  |  |  |  |  |  |  |  |  | **0.04** |
| **rs11168287** | **(IVS2+8206G>A)** | |  |  |  |  |  |  |  |  |  |  |  |
| (VDR-77) | GG | 184 | 23.7 |  | 268 | 26.0 |  | 1.00 |  |  |  |  |  |
|  | AG | 426 | 54.8 |  | 526 | 51.0 |  | 1.25 | 0.99 | **-** | 1.58 |  |  |
|  | AA | 167 | 21.5 |  | 237 | 23.0 |  | 1.09 | 0.82 | **-** | 1.45 |  |  |
|  |  |  |  |  |  |  |  |  |  |  |  |  | 0.50 |
| **rs4760648** | **(IVS2-4108C>T)** |  |  |  |  |  |  |  |  |  |  |  |  |
| (VDR-42) | CC | 289 | 37.2 |  | 326 | 31.6 |  | 1.00 |  |  |  |  |  |
|  | CT | 374 | 48.1 |  | 507 | 49.2 |  | 0.81 | 0.65 | - | 1.00 |  |  |
|  | TT | 114 | 14.7 |  | 198 | 19.2 |  | 0.64 | 0.48 | - | 0.85 |  |  |
|  |  |  |  |  |  |  |  |  |  |  |  |  | **0.002** |
| **rs2853564** | **(IVS2-1930A>G)** | |  |  |  |  |  |  |  |  |  |  |  |
| (VDR-39) | AA | 286 | 36.9 |  | 434 | 42.0 |  | 1.00 |  |  |  |  |  |
|  | AG | 378 | 48.7 |  | 456 | 44.1 |  | 1.29 | 1.05 | - | 1.59 |  |  |
|  | GG | 112 | 14.4 |  | 144 | 13.9 |  | 1.29 | 0.96 | - | 1.74 |  |  |
|  |  |  |  |  |  |  |  |  |  |  |  |  | **0.03** |
| **rs2254210** | **(IVS3-816G>A)** |  |  |  |  |  |  |  |  |  |  |  |  |
| (VDR-36) | GG | 324 | 41.7 |  | 483 | 46.8 |  | 1.00 |  |  |  |  |  |
|  | AG | 355 | 45.7 |  | 443 | 42.9 |  | 1.21 | 0.99 | - | 1.48 |  |  |
|  | AA | 98 | 12.6 |  | 107 | 10.4 |  | 1.40 | 1.02 | - | 1.93 |  |  |
|  |  |  |  |  |  |  |  |  |  |  |  |  | **0.02** |
| **rs2228570** | **(Ex4+4T>C)** |  |  |  |  |  |  |  |  |  |  |  |  |
| *FokI* | TT | 249 | 35.4 |  | 297 | 32.9 |  | 1.00 |  |  |  |  |  |
| (VDR-04) | CT | 331 | 47.0 |  | 426 | 47.2 |  | 0.91 | 0.72 | **-** | 1.14 |  |  |
|  | CC | 124 | 17.6 |  | 179 | 19.8 |  | 0.82 | 0.61 | **-** | 1.10 |  |  |
|  |  |  |  |  |  |  |  |  |  |  |  |  | 0.18 |
| **rs2239186** | **(IVS4+3341A>G)** |  |  |  |  |  |  |  |  |  |  |  |  |
| (VDR-35) | AA | 447 | 57.5 |  | 581 | 56.2 |  | 1.00 |  |  |  |  |  |
|  | AG | 295 | 38.0 |  | 388 | 37.6 |  | 1.00 | 0.82 | **-** | 1.22 |  |  |
|  | GG | 35 | 4.5 |  | 64 | 6.2 |  | 0.71 | 0.46 | **-** | 1.11 |  |  |
|  |  |  |  |  |  |  |  |  |  |  |  |  | 0.34 |
| **rs3782905** | **(IVS4+6584G>C)** |  |  |  |  |  |  |  |  |  |  |  |  |
| (VDR-40) | GG | 373 | 48.0 |  | 514 | 49.7 |  | 1.00 |  |  |  |  |  |
|  | CG | 352 | 45.3 |  | 425 | 41.1 |  | 1.15 | 0.94 | **-** | 1.41 |  |  |
|  | CC | 52 | 6.7 |  | 95 | 9.2 |  | 0.71 | 0.49 | **-** | 1.04 |  |  |
|  |  |  |  |  |  |  |  |  |  |  |  |  | 0.70 |
| **rs3819545** | **(IVS4-6046A>G)** |  |  |  |  |  |  |  |  |  |  |  |  |
| (VDR-101) | AA | 275 | 35.4 |  | 337 | 32.6 |  | 1.00 |  |  |  |  |  |
|  | AG | 394 | 50.7 |  | 524 | 50.7 |  | 0.95 | 0.77 | **-** | 1.18 |  |  |
|  | GG | 108 | 13.9 |  | 172 | 16.7 |  | 0.80 | 0.60 | **-** | 1.08 |  |  |
|  |  |  |  |  |  |  |  |  |  |  |  |  | 0.18 |
| **rs2189480** | **(IVS4-4868G>T)** |  |  |  |  |  |  |  |  |  |  |  |  |
| (VDR-32) | GG | 294 | 37.8 |  | 390 | 37.7 |  | 1.00 |  |  |  |  |  |
|  | GT | 385 | 49.5 |  | 498 | 48.1 |  | 1.02 | 0.83 | **-** | 1.25 |  |  |
|  | TT | 98 | 12.6 |  | 147 | 14.2 |  | 0.88 | 0.65 | **-** | 1.20 |  |  |
|  |  |  |  |  |  |  |  |  |  |  |  |  | 0.57 |
| **rs886441** | **(IVS4-4004A>G)** |  |  |  |  |  |  |  |  |  |  |  |  |
| (VDR-92) | AA | 464 | 59.7 |  | 672 | 65.1 |  | 1.00 |  |  |  |  |  |
|  | AG/GG | 313 | 40.3 |  | 361 | 34.9 |  | 1.24 | 1.02 | - | 1.51 |  |  |
|  |  |  |  |  |  |  |  |  |  |  |  |  |  |
|  |  |  |  |  |  |  |  |  |  |  |  |  | **0.02** |
| **rs12717991** | **(IVS4-166C>T)** |  |  |  |  |  |  |  |  |  |  |  |  |
| (VDR-97) | CC | 293 | 37.8 |  | 336 | 32.5 |  | 1.00 |  |  |  |  |  |
|  | CT | 355 | 45.7 |  | 501 | 48.5 |  | 0.84 | 0.68 | **-** | 1.04 |  |  |
|  | TT | 128 | 16.5 |  | 196 | 19.0 |  | 0.79 | 0.60 | **-** | 1.05 |  |  |
|  |  |  |  |  |  |  |  |  |  |  |  |  | 0.07 |
| **rs2239179** | **(IVS5+1064T>C)** |  |  |  |  |  |  |  |  |  |  |  |  |
| (VDR-34) | TT | 286 | 36.8 |  | 381 | 36.8 |  | 1.00 |  |  |  |  |  |
|  | CT | 384 | 49.4 |  | 507 | 49.0 |  | 0.99 | 0.81 | **-** | 1.22 |  |  |
|  | CC | 107 | 13.8 |  | 146 | 14.1 |  | 0.92 | 0.68 | **-** | 1.24 |  |  |
|  |  |  |  |  |  |  |  |  |  |  |  |  | 0.63 |
| **rs2239180** | **(IVS5+2784C>G)** |  |  |  |  |  |  |  |  |  |  |  |  |
| (VDR-108) | CC | 636 | 81.9 |  | 854 | 82.7 |  | 1.00 |  |  |  |  |  |
|  | CG/GG | 141 | 18.1 |  | 179 | 17.3 |  | 1.06 | 0.83 | **-** | 1.37 |  |  |
|  |  |  |  |  |  |  |  |  |  |  |  |  |  |
|  |  |  |  |  |  |  |  |  |  |  |  |  | 0.77 |
| **rs2107301** | **(IVS5+3260G>A)** |  |  |  |  |  |  |  |  |  |  |  |  |
| (VDR-81) | GG | 384 | 49.7 |  | 481 | 46.6 |  | 1.00 |  |  |  |  |  |
|  | AG | 319 | 41.3 |  | 447 | 43.3 |  | 0.92 | 0.75 | **-** | 1.12 |  |  |
|  | AA | 69 | 8.9 |  | 105 | 10.2 |  | 0.83 | 0.59 | **-** | 1.17 |  |  |
|  |  |  |  |  |  |  |  |  |  |  |  |  | 0.23 |
| **rs2239182** | **(IVS5+3419T>C)** |  |  |  |  |  |  |  |  |  |  |  |  |
| (VDR-84) | TT | 225 | 29.0 |  | 299 | 28.9 |  | 1.00 |  |  |  |  |  |
|  | CT | 391 | 50.3 |  | 526 | 50.9 |  | 0.96 | 0.77 | **-** | 1.20 |  |  |
|  | CC | 161 | 20.7 |  | 209 | 20.2 |  | 0.96 | 0.73 | **-** | 1.27 |  |  |
|  |  |  |  |  |  |  |  |  |  |  |  |  | 0.76 |
| **rs2248098** | **(IVS5-1885A>G)** |  |  |  |  |  |  |  |  |  |  |  |  |
| (VDR-109) | AA | 234 | 30.1 |  | 285 | 27.5 |  | 1.00 |  |  |  |  |  |
|  | AG | 371 | 47.7 |  | 529 | 51.1 |  | 0.85 | 0.68 | **-** | 1.06 |  |  |
|  | GG | 172 | 22.1 |  | 221 | 21.4 |  | 0.92 | 0.70 | **-** | 1.21 |  |  |
|  |  |  |  |  |  |  |  |  |  |  |  |  | 0.47 |
| **rs11574077** | **(IVS5-1456T>C)** |  |  |  |  |  |  |  |  |  |  |  |  |
| (VDR-94) | TT | 701 | 90.2 |  | 938 | 90.6 |  | 1.00 |  |  |  |  |  |
|  | CT/CC | 76 | 9.8 |  | 97 | 9.4 |  | 0.94 | 0.68 | **-** | 1.30 |  |  |
|  |  |  |  |  |  |  |  |  |  |  |  |  |  |
|  |  |  |  |  |  |  |  |  |  |  |  |  | 0.71 |
| **rs1544410** | **(IVS10+283C>T)** |  |  |  |  |  |  |  |  |  |  |  |  |
| *BsmI* | CC | 315 | 40.6 |  | 412 | 39.8 |  | 1.00 |  |  |  |  |  |
| (VDR-08) | CT | 358 | 46.1 |  | 481 | 46.5 |  | 0.96 | 0.78 | **-** | 1.18 |  |  |
|  | TT | 103 | 13.3 |  | 142 | 13.7 |  | 0.89 | 0.65 | **-** | 1.20 |  |  |
|  |  |  |  |  |  |  |  |  |  |  |  |  | 0.44 |
| **rs3847987** | **(Ex11+721C>A)** |  |  |  |  |  |  |  |  |  |  |  |  |
| (VDR-64) | CC | 620 | 79.8 |  | 828 | 80.1 |  | 1.00 |  |  |  |  |  |
|  | AC/AA | 157 | 20.2 |  | 206 | 19.9 |  | 1.02 | 0.80 | **-** | 1.29 |  |  |
|  |  |  |  |  |  |  |  |  |  |  |  |  |  |
|  |  |  |  |  |  |  |  |  |  |  |  |  | 0.99 |
| **rs12721364** | **(*7098G>A)** |  |  |  |  |  |  |  |  |  |  |  |  |
| (VDR-98) | GG | 536 | 69.0 |  | 694 | 67.1 |  | 1.00 |  |  |  |  |  |
|  | AG/AA | 241 | 31.0 |  | 341 | 32.9 |  | 0.92 | 0.75 | **-** | 1.12 |  |  |
|  |  |  |  |  |  |  |  |  |  |  |  |  | 0.60 |
| Adjusted for sex, center, age (continuous), and smoking status (ever, never) | | | | | | | | | | | | | |
