# Supplementary figures and images for: Analysis of SNPs and Haplotypes in Vitamin D Pathway Genes and Renal Cancer Risk
Source: PLoS One. 2009 Sep 15;4(9):e7013. doi: 10.1371/journal.pone.0007013 (PMC2737618; doi:10.1371/journal.pone.0007013)

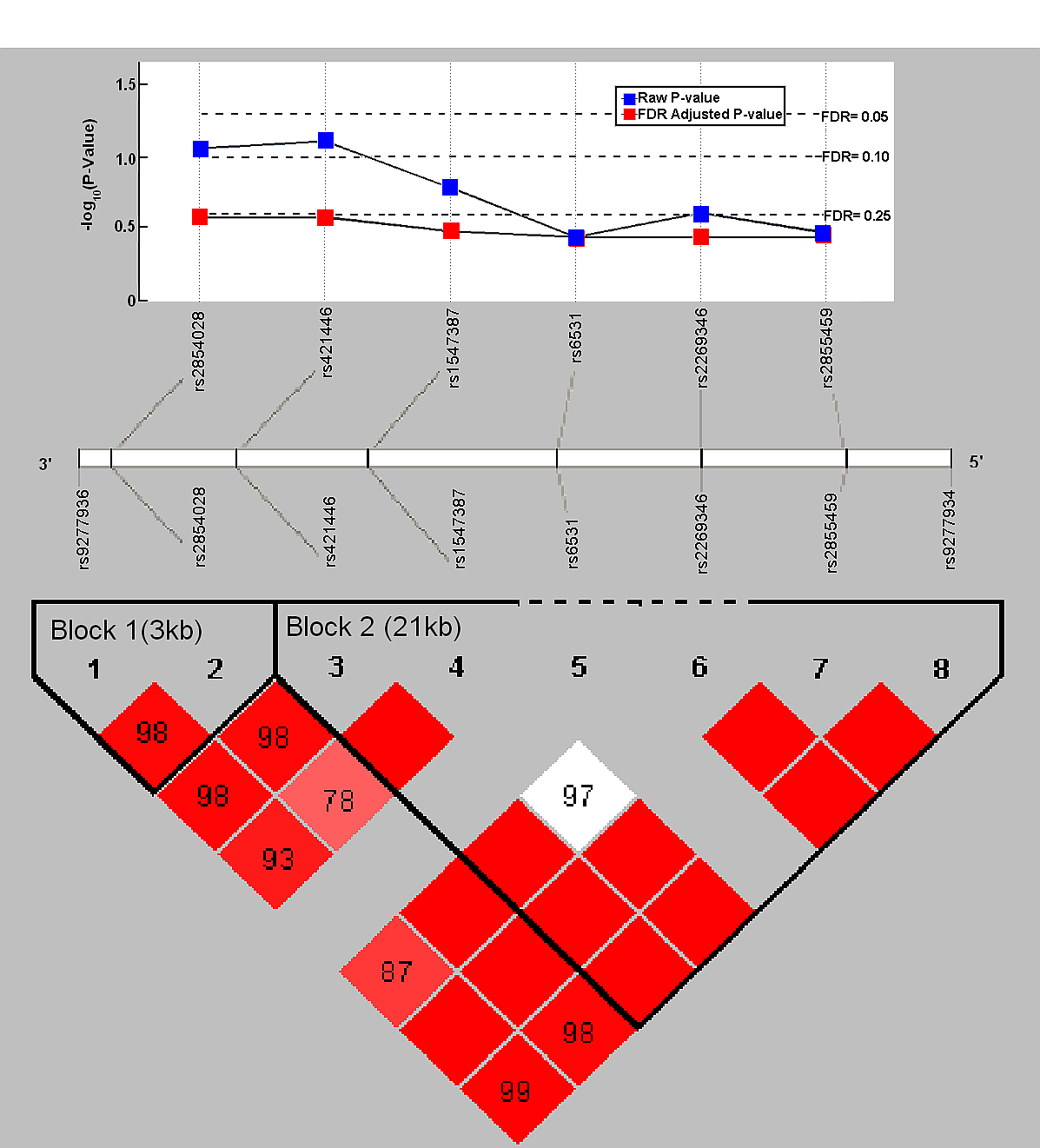

Supplement: Figure S1 — RXRB HaploWalk and Haploview analysis. Top: HaploWalk analysis identified no chromosomal regions of interest in the retinoid-X-receptor-beta (RXRB) gene that were significant at an FDR of 10%. The Min-P test for this gene was not statistically significant (p-value = 0.20). Bottom: Haploview analysis identified two haplotype blocks; none were associated with RCC risk when analyzed in Haplostats. (0.24 MB TIF) [file pone.0007013.s002.tif]

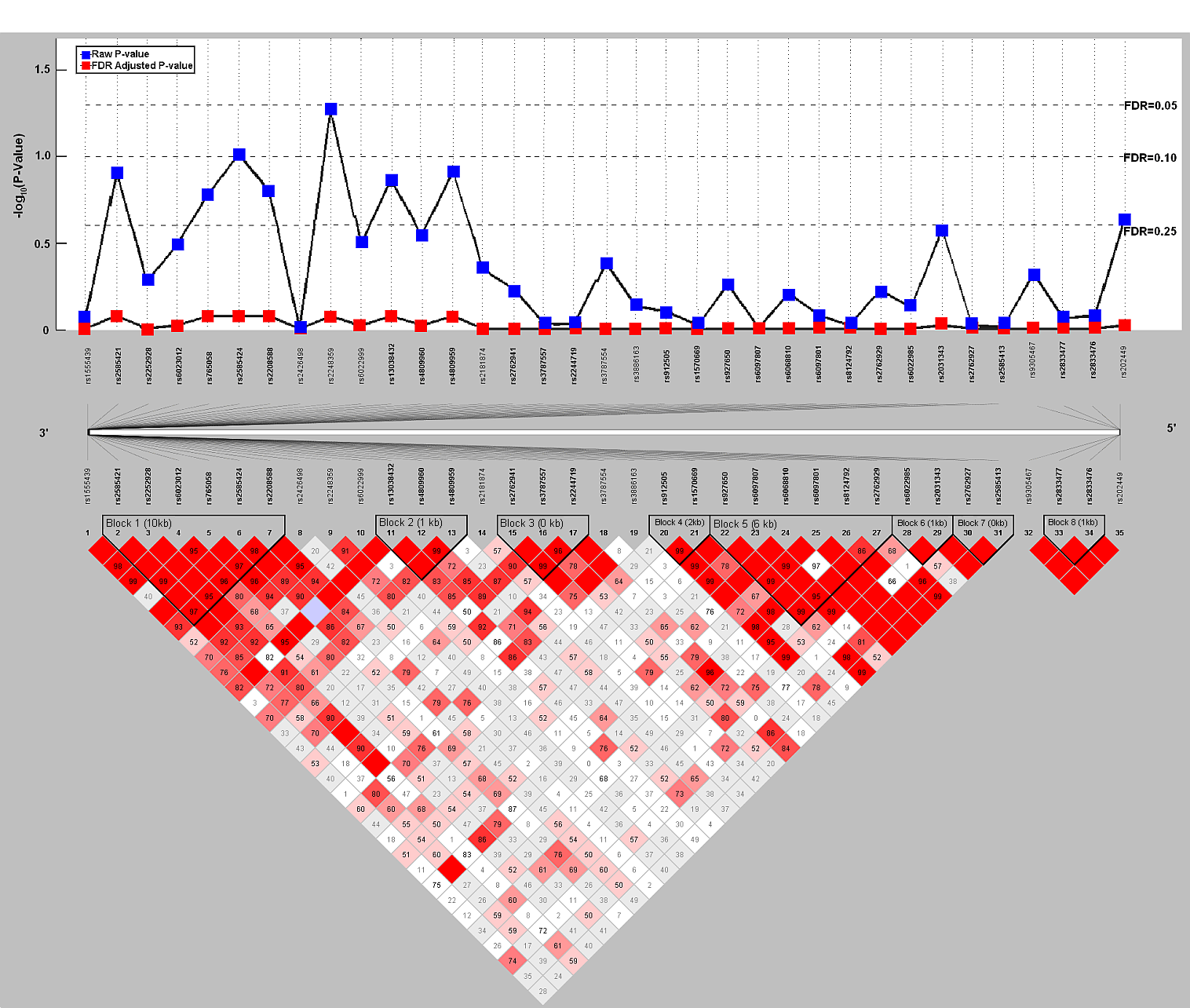

Supplement: Figure S2 — CYP24A1 HaploWalk and Haploview analysis. Top: HaploWalk analysis identified no chromosomal regions of interest in the cytochrome P450, family 24, subfamily A, polypeptide 1 (CYP24A1) gene that were significant at an FDR of 10%. The Min-P test for this gene was not statistically significant (p-value = 0.63). Bottom: Haploview analysis identified several haplotype blocks; none were associated with RCC risk when analyzed in Haplostats. (0.76 MB TIF) [file pone.0007013.s003.tif]

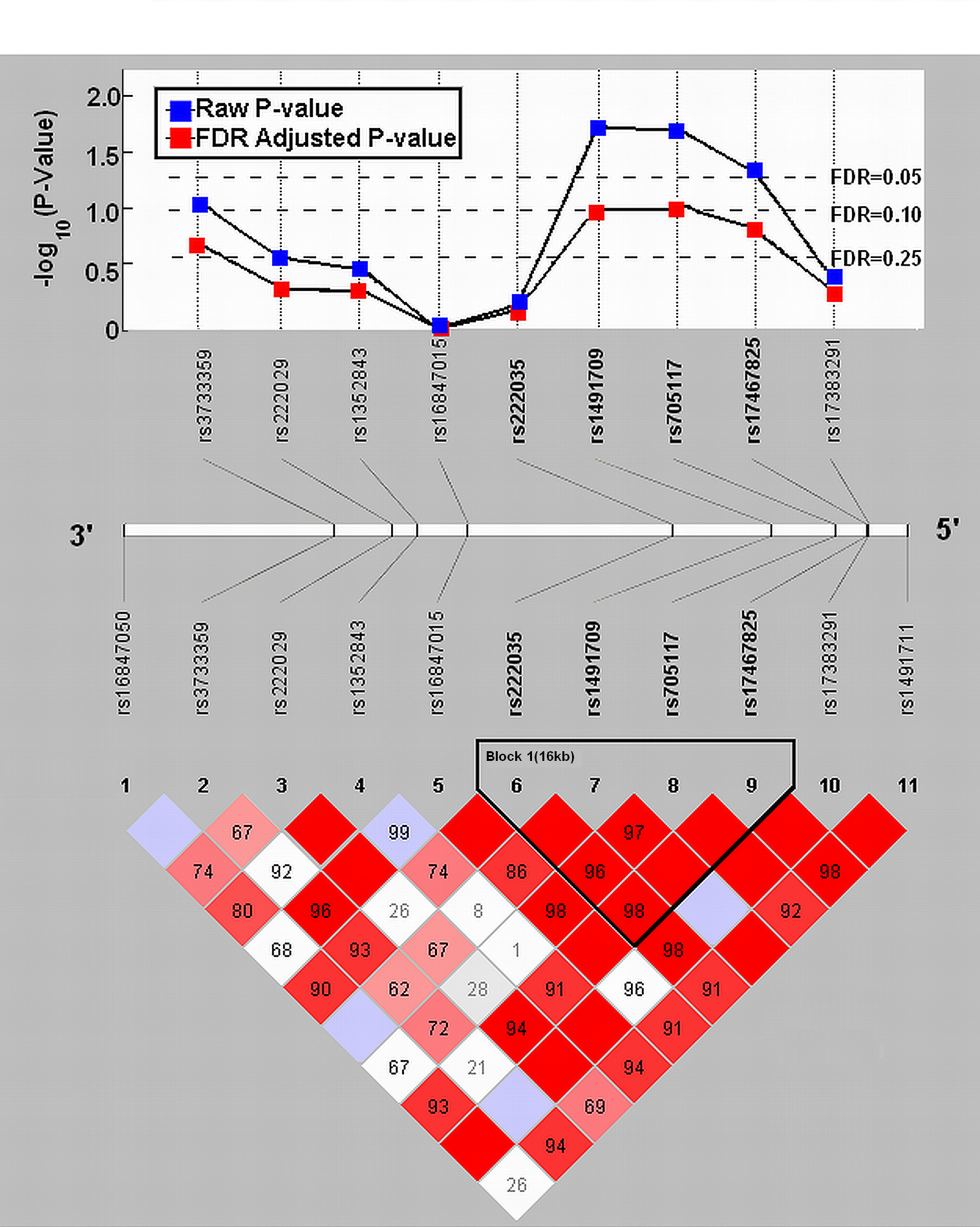

Supplement: Figure S3 — GC HaploWalk and Haploview analysis. Top: HaploWalk analysis identified no chromosomal regions of interest in the group specific component (GC) vitamin D binding protein that were significant at an FDR of 10%. The Min-P test for this gene was not statistically significant (p-value = 0.25). Bottom: Haploview analysis identified one haplotype block which was not associated with RCC risk when analyzed in Haplostats. (2.86 MB TIF) [file pone.0007013.s004.tif]

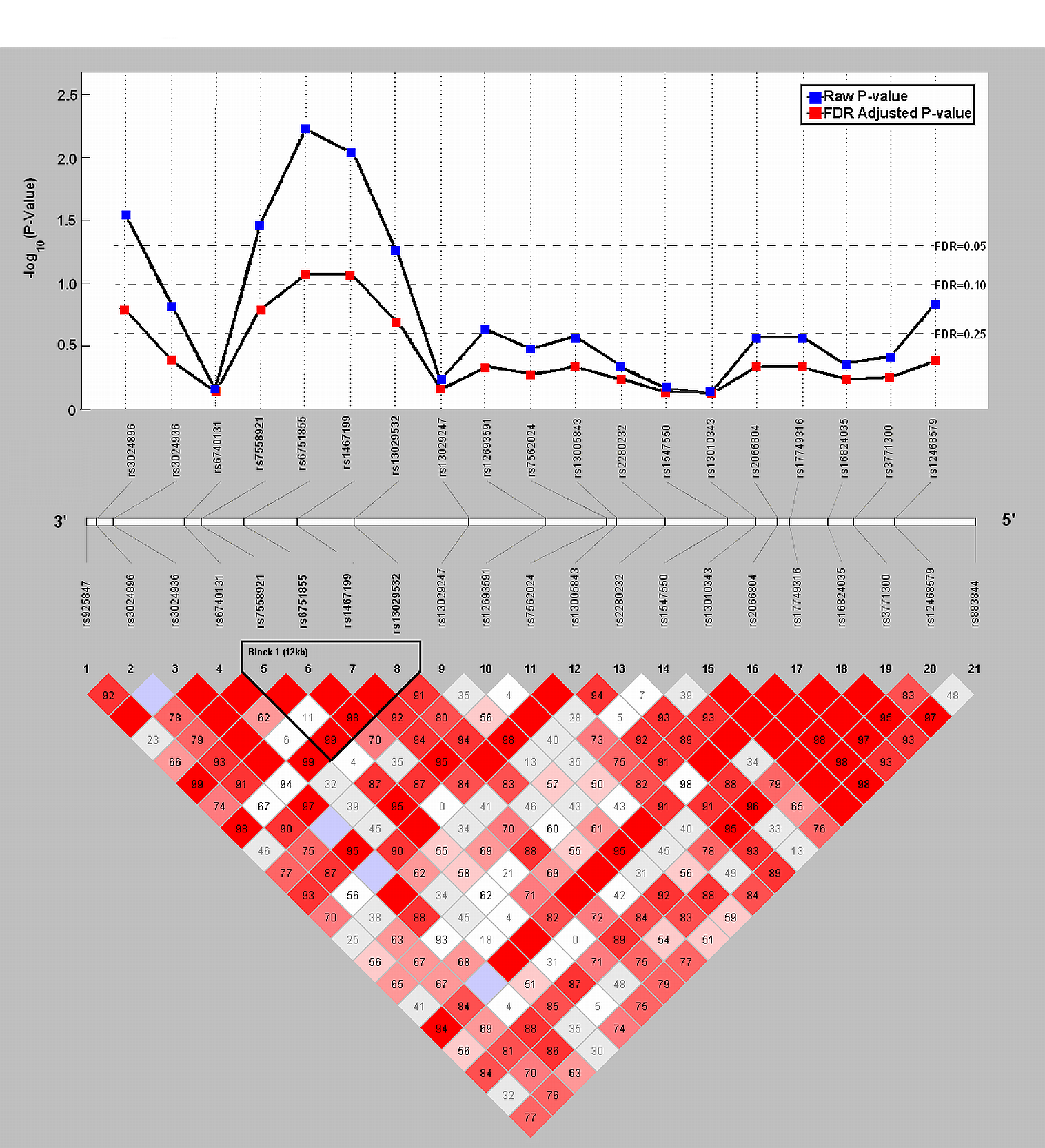

Supplement: Figure S4 — STAT1 HaploWalk and Haploview analysis. Top: HaploWalk analysis identified a single chromosomal region of interest in the signal transducer and activator of transcription 1 (STAT1) gene that was significant at an FDR of 10%. This region included SNPs 3′- rs3755821, rs6751855, rs1467199, rs13029532 -5′. The Min-P test for this gene was not statistically significant (p-value = 0.14). Bottom: Haploview analysis also identified one haplotype block within the same chromosomal region of interest. Haplostats analysis adjusted for age, sex, study center, and smoking habit showed that subjects with the CGGA haplotype had an increased risk of RCC (OR = 1.21; 95% CI = 1.01–1.45; p-value = 0.04) compared to subjects with the referent haplotype, GACA. (1.76 MB TIF) [file pone.0007013.s005.tif]

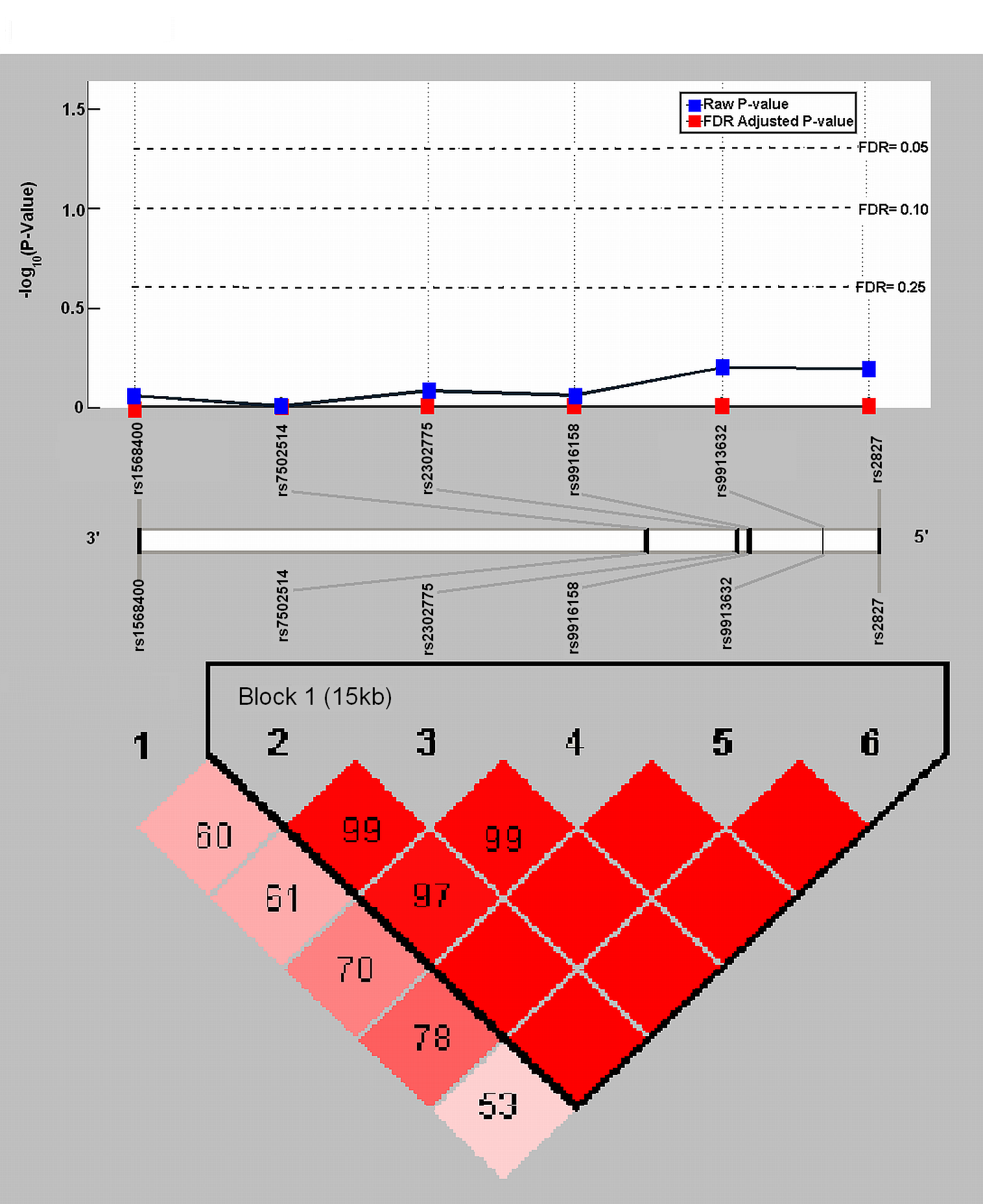

Supplement: Figure S5 — THRAP4 HaploWalk and Haploview analysis. Top: HaploWalk analysis identified no chromosomal regions of interest in the thyroid hormone receptor associated protein 4 (THRAP4) gene that were significant at an FDR of 10%. The Min-P test for this gene was not statistically significant (p-value = 0.94). Bottom: Haploview analysis identified one haplotype block which was not associated with RCC risk when analyzed in Haplostats. (1.42 MB TIF) [file pone.0007013.s006.tif]

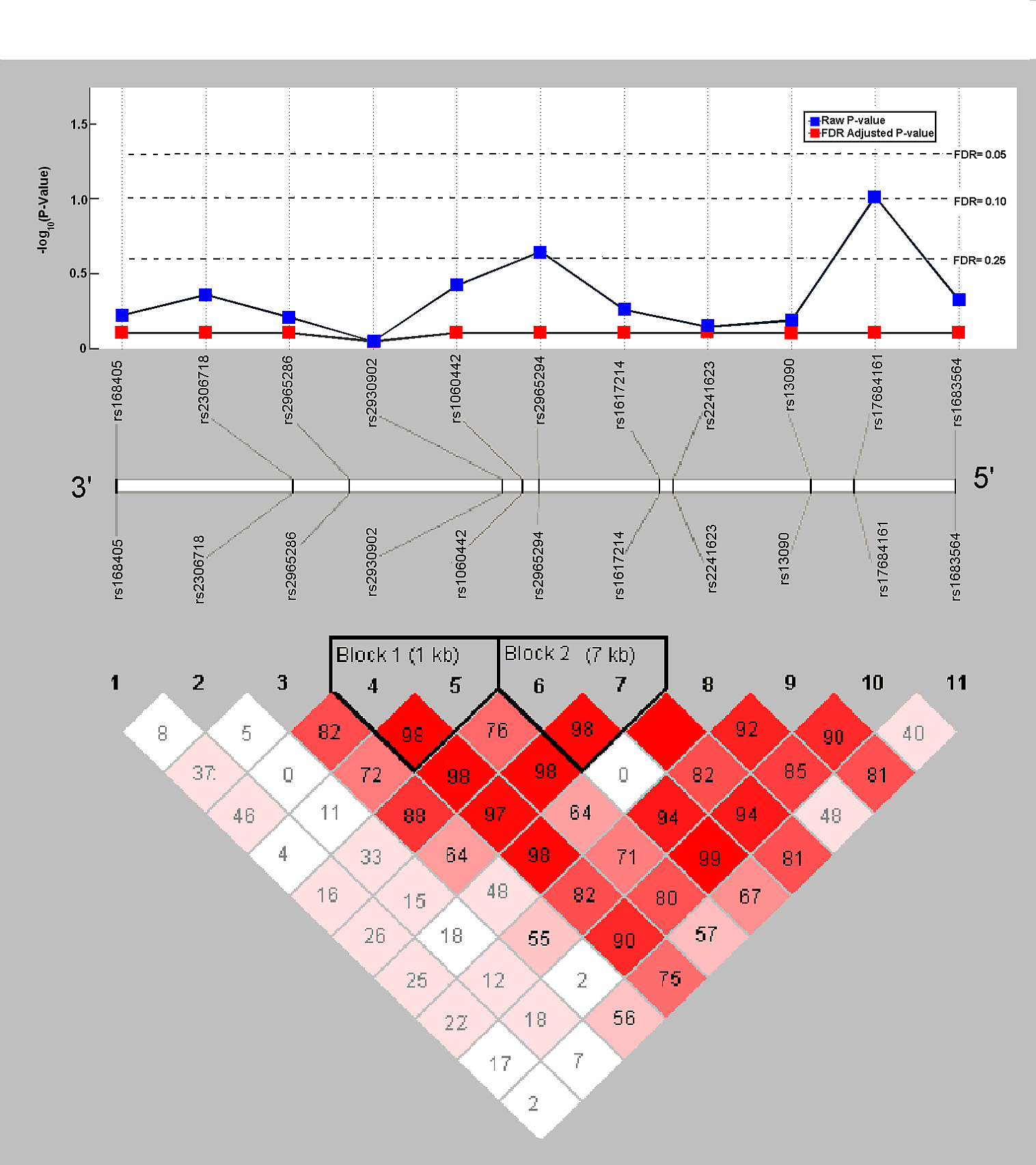

Supplement: Figure S6 — THRAP5 HaploWalk and Haploview analysis. Top: HaploWalk analysis identified no chromosomal regions of interest in the thyroid hormone receptor associated protein 5 (THRAP5) gene that were significant at an FDR of 10%. The Min-P test for this gene was not statistically significant (p-value = 0.38). Bottom: Haploview analysis identified several haplotype blocks; none were associated with RCC risk when analyzed in Haplostats. (0.64 MB TIF) [file pone.0007013.s007.tif]
